# Large global variations in measured airborne metal concentrations driven by anthropogenic sources

**DOI:** 10.1038/s41598-020-78789-y

**Published:** 2020-12-11

**Authors:** Jacob McNeill, Graydon Snider, Crystal L. Weagle, Brenna Walsh, Paul Bissonnette, Emily Stone, Ihab Abboud, Clement Akoshile, Nguyen Xuan Anh, Rajasekhar Balasubramanian, Jeffrey R. Brook, Craig Coburn, Aaron Cohen, Jinlu Dong, Graham Gagnon, Rebecca M. Garland, Kebin He, Brent N. Holben, Ralph Kahn, Jong Sung Kim, Nofel Lagrosas, Puji Lestari, Yang Liu, Farah Jeba, Khaled Shaifullah Joy, J. Vanderlei Martins, Amit Misra, Leslie K. Norford, Eduardo J. Quel, Abdus Salam, Bret Schichtel, S. N. Tripathi, Chien Wang, Qiang Zhang, Michael Brauer, Mark D. Gibson, Yinon Rudich, Randall V. Martin

**Affiliations:** 1 Department of Chemistry, Dalhousie University, Halifax, Canada; 2 Department of Energy, Environmental and Chemical Engineering, Washington University in St. Louis, St. Louis, MO USA; 3 Department of Physics and Atmospheric Science, Dalhousie University, Halifax, Canada; 4 Environment and Climate Change Canada, Downsview, ON Canada; 5 Department of Physics, University of Ilorin, Ilorin, Nigeria; 6 Institute of Geophysics, Vietnam Academy of Science and Technology, Hanoi, Vietnam; 7 Department of Civil and Environmental Engineering, National University of Singapore, Singapore, Singapore; 8 Department of Public Health Sciences, University of Toronto, Toronto, ON M5S 1A8 Canada; 9 Department of Geography and Environment, University of Lethbridge, Lethbridge, AB Canada; 10 Health Effects Institute, 75 Federal Street Suite 1400, Boston, MA 02110-1817 USA; 11 School of Environment, Tsinghua University, Beijing, China; 12 Department of Civil and Resource Engineering, Dalhousie University, Halifax, Canada; 13 Council for Scientific and Industrial Research (CSIR), Pretoria, South Africa; 14 Unit for Environmental Sciences and Management, North-West University, Potchefstroom, South Africa; 15 Department of Geography, Geo-Informatics and Meteorology, University of Pretoria, Pretoria, South Africa; 16 Department of Earth System Science, Tsinghua University, Beijing, China; 17 Earth Science Division, NASA Goddard Space Flight Center, Greenbelt, MD USA; 18 Department of Community Health and Epidemiology, Dalhousie University, Halifax, Canada; 19 Manila Observatory, Ateneo de Manila University Campus, Quezon City, Philippines; 20 Faculty of Civil and Environmental Engineering, Bandung Institute of Technology (ITB), JL. Ganesha No.10, Bandung, 40132 Indonesia; 21 Gangarosa Department of Environmental Health, Rollins School of Public Health, Emory University, Atlanta, GA 30322 USA; 22 Department of Chemistry, Faculty of Science, University of Dhaka, Dhaka, 1000 Bangladesh; 23 Department of Physics and Joint Center for Earth Systems Technology, University of Maryland, Baltimore County, Baltimore, MD USA; 24 Center for Environmental Science and Engineering, Department of Civil Engineering, Indian Institute of Technology Kanpur, Kanpur, India; 25 Department of Architecture, Massachusetts Institute of Technology, Cambridge, MA 02139 USA; 26 UNIDEF (CITEDEF-CONICET), Juan B. de la Salle 4397 – B1603ALO Villa Martelli, Buenos Aires, Argentina; 27 Cooperative Institute for Research in the Atmosphere, Colorado State University, Fort Collins, CO USA; 28 Laboratoire d’Aerologie, CNRS/UPS, 14 Avenue Edouard Belin, Toulouse, France; 29 School of Population and Public Health, University of British Columbia, Vancouver, BC Canada; 30 AirPhoton, LLC., Baltimore, MD USA; 31 Department of Earth and Planetary Sciences, Weizmann Institute, 76100 Rehovot, Israel

**Keywords:** Atmospheric science, Atmospheric chemistry

## Abstract

Globally consistent measurements of airborne metal concentrations in fine particulate matter (PM_2.5_) are important for understanding potential health impacts, prioritizing air pollution mitigation strategies, and enabling global chemical transport model development. PM_2.5_ filter samples (N ~ 800 from 19 locations) collected from a globally distributed surface particulate matter sampling network (SPARTAN) between January 2013 and April 2019 were analyzed for particulate mass and trace metals content. Metal concentrations exhibited pronounced spatial variation, primarily driven by anthropogenic activities. PM_2.5_ levels of lead, arsenic, chromium, and zinc were significantly enriched at some locations by factors of 100–3000 compared to crustal concentrations. Levels of metals in PM_2.5_ and PM_10_ exceeded health guidelines at multiple sites. For example, Dhaka and Kanpur sites exceeded the US National Ambient Air 3-month Quality Standard for lead (150 ng m^−3^). Kanpur, Hanoi, Beijing and Dhaka sites had annual mean arsenic concentrations that approached or exceeded the World Health Organization’s risk level for arsenic (6.6 ng m^−3^). The high concentrations of several potentially harmful metals in densely populated cites worldwide motivates expanded measurements and analyses.

## Introduction

Many regions of the world far exceed the World Health Organization (WHO) air quality guidelines for ambient fine particulate matter (PM_2.5_)^1,2^ air pollution levels, with substantial impacts on human health^3^. The Global Burden of Disease estimated 3 million deaths (9% of all deaths) and 80 million years of lost healthy life were attributable to exposure to outdoor PM_2.5_ globally in 2017^4^. Nonetheless, ground-based monitoring of PM_2.5_ mass concentration is inadequate for exposure assessment^5^, and the select studies of PM_2.5_ chemical composition measurements^6,7^ are even sparser. In some locations, the majority of metal concentration measurements made to date have been of the PM_10_ fraction^8–10^. Global observations of PM_2.5_ mass concentration and composition can inform aerosol model development and exposure assessment, improve understanding of emission sources and help prioritize mitigation policies^11^ to reduce health impacts.

The relationship between PM_2.5_ and human health^12,13^ including the association of PM_2.5_ with cardiovascular disease^14^, respiratory disease^15^, cancer^16^, and type 2 diabetes^17^ has become better understood over the last few decades. However, more analysis of these relationships is needed before effects of particular components (specifically trace metals) are well understood at a global scale. The oxidative potential of PM_2.5_ is related to its metal content^6^ (as well as its carbon content^18^) and increased abundance of redox-active elements can induce oxidative stress^19–22^. Increased cardiovascular disease rates have been associated with exposure to enhanced relative concentrations of K, Al, Ni, Zn and V^14,23^ and mortality risks have been associated with preferential bioaccumulation of heavy metals such as As, Pb, and Al^24^. Further, many metals have known health effects, such as As, Cd, and Cr, which are classified by the WHO’s International Agency for Research on Cancer (IARC) as known human carcinogens (IARC Group 1)^25^, and Pb, which is associated with impaired cognitive function^26^. Measurements of PM_2.5_ composition are needed to assess the global distribution of these deleterious metals in fine particulate matter, as they could pose health risks to populations living in areas of high PM_2.5_ pollution.

Ground-based elemental composition can also provide information about airborne PM_2.5_ sources. For example, K has associations with wood burning^27–29^, Zn can be linked with traffic through tire wear^30^, and V derives mainly from heavy fuel oil combustion such as from shipping^31,32^. Coal is a source of multiple potentially harmful elements such as Pb, Cr, Mn, As, and Se^31,33^ whereas non-ferrous metal production is a large source of As, Cd, and Zn^31^. Vehicle traffic contributes a mix of elements including the heavy metals Ba, Zn, and Pb, as well as the crustal components Fe, Al, Mg, and Ti^27,34^. To our knowledge, no other global network has measured the trace metal concentrations in PM_2.5_. These observations are needed to better understand particulate matter sources and loadings, to assess emerging emission inventories and evaluate spatially-resolved concentration predictions made by chemical transport models on a global scale^35,36^ and to understand local and regional impacts of emission sources.

The ground-based Surface PARTiculate mAtter Network (SPARTAN; http://www.spartan-network.org) is building up a long-term data set measuring PM concentration at globally distributed sites^37^ and provides new data to evaluate PM composition. In this study, we investigate the trace metal composition of PM_2.5_, supplemented by coarse PM (PM_c_ = PM_10-2.5_) and PM_10_ data, in ambient air samples from SPARTAN sites around the world. Our focus is on PM_2.5_ due to its importance for health.

## Results and discussion

**Figure 1:**
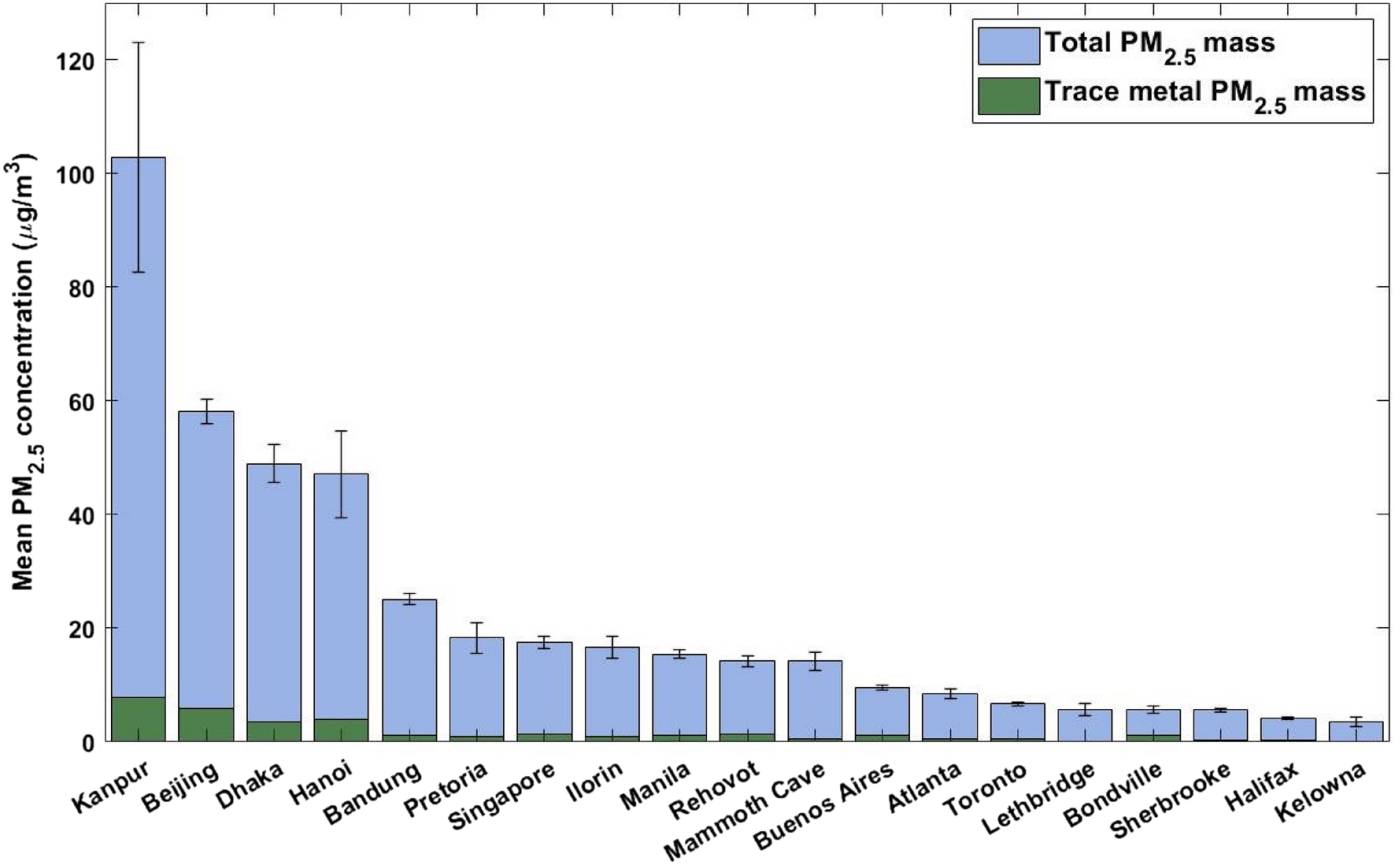
Mean PM_2.5_ mass concentrations at SPARTAN sites with standard error bars shown. Overlaid green bars show total measured trace metal mean mass concentrations for each site.

The mean PM_2.5_ mass concentrations at each SPARTAN site are shown in Fig. 1, with standard error bars (SE = $\sigma/\sqrt{n}$, σ = sample standard deviation, n = number of samples) for each site mean. Of the 19 SPARTAN sites, Kanpur had the highest mean PM_2.5_ levels at 102.8 ± 20.2 (SE) µg/m^3^, followed by Beijing at 58.1 ± 2.2 µg/m^3^, then Dhaka (49.0 ± 3.3 µg/m^3^) and Hanoi (47.1 ± 7.6 µg/m^3^).

Sites located in urban areas situated outside of North America generally had the highest levels of PM_2.5_. The one notable exception was the Mammoth Cave site, which had the 11th highest PM_2.5_ levels. This can be explained through two aspects of the sampling; firstly, the site is located in a heavily wooded national park; sampling occurred in the summer when carbonaceous emissions are highest from biogenic sources, and secondary organic aerosol is prevalent^38^. Secondly, the site has the second-lowest percentage of its PM_2.5_ mass present in the measured trace metal mass (4%), indicating other sources (i.e. biogenic) were dominant. Sites located in Canada generally had the lowest levels of PM_2.5_, which is consistent with their intended categorization as low-PM environments.

Table 1 details the mean mass concentrations of 15 trace metals analyzed in PM_2.5_ samples from SPARTAN sites. Elements above ICP-MS detection limits (detailed in supplemental Table S1) for > 10% of samples were considered; Li, Co, Ag, and Ce did not satisfy this requirement, and as such are not discussed here. Individual elements were examined to understand their distributions across sites (a detailed breakdown across sites can be found in the supplemental section S1, with a corresponding table of standard deviations in supplemental Table S4). Elemental breakdowns for the PM_10_ fraction are found in supplemental Table S7, while relative concentrations of each trace element in PM_2.5_ can be found in supplemental Table S8.

**Table 1:** Full elemental breakdown of mean mass concentrations of trace metals in PM_2.5_ at SPARTAN sites.

	PM_2.5_	K	Mg	P	Ti	V	Cr	Mn	Fe	Cu	Zn	As	Se	Cd	Ba	Pb
Mammoth Cave	14.2	74.8	28.0	55.7	1.01	0.78	1.20	1.84	83.2	3.68	8.5	0.26	0.28	0.05	3.12	0.90
Atlanta	8.6	27.8	11.5	105.3	1.24	0.18	4.20	0.71	51.5	3.67	9.3	0.56	0.54	0.02	5.41	1.08
Bandung	25.1	432.1	18.3	23.9	1.73	0.31	2.12	2.80	78.2	3.28	23.7	0.60	0.23	0.32	2.57	34.62
Beijing	58.1	962.6	177.3	151.1	11.18	2.30	4.39	23.84	394.7	26.73	101.5	7.12	67.40	3.29	21.81	41.30
Bondville	5.7	64.1	34.1	162.3	1.39	0.13	3.77	1.59	37.0	3.11	16.7	0.65	0.55	0.12	2.15	1.54
Buenos Aires	9.6	152.1	30.4	30.3	1.70	2.48	1.10	2.52	94.0	5.17	20.8	0.43	0.36	0.30	5.08	10.47
Dhaka	49.0	876.0	47.9	23.0	3.94	6.98	8.00	25.40	167.7	11.69	498.4	6.33	5.38	7.35	12.53	279.72
Halifax	4.2	40.0	17.8	1.1	0.25	0.23	0.39	0.36	10.8	0.76	3.7	0.14	0.07	0.00	0.75	0.49
Hanoi	47.1	1293.8	84.5	36.6	5.21	2.10	2.28	80.05	282.2	14.10	1178.8	8.11	3.00	4.25	7.13	141.04
Ilorin	16.6	355.6	15.5	5.1	0.88	0.61	47.96	4.51	182.4	0.94	12.9	0.22	0.14	0.06	0.90	4.27
Kanpur	102.8	3047.1	74.4	340.1	5.60	2.21	19.41	9.89	168.0	8.78	119.5	15.29	10.60	12.88	3.99	209.33
Kelowna	3.5	34.1	2.4	1.6	0.32	0.14	0.32	0.41	17.0	0.55	1.4	0.19	0.04	0.01	0.75	0.29
Lethbridge	6.2	56.3	6.4	1.5	0.30	0.03	0.25	0.68	18.5	0.76	1.9	0.17	0.14	0.03	0.85	0.38
Manila	15.4	253.3	20.3	34.4	1.30	2.32	2.97	3.17	111.2	2.93	29.4	0.33	0.96	0.25	2.31	5.89
Pretoria	18.3	220.0	14.8	37.5	1.66	0.44	0.80	5.78	105.2	2.28	27.5	1.00	0.54	0.10	2.54	4.88
Sherbrooke	5.7	48.3	5.0	4.3	0.45	0.03	0.20	0.92	16.7	0.73	4.2	0.26	0.10	0.02	0.58	1.08
Rehovot	15.4	135.2	79.9	11.4	2.18	2.95	1.56	2.82	123.5	3.22	12.8	0.26	0.34	0.09	3.69	4.64
Singapore	17.5	344.9	24.2	13.4	1.66	37.92	0.47	7.76	89.9	5.57	110.1	0.48	0.71	0.12	3.80	3.54
Toronto	6.7	71.0	12.0	9.7	0.83	0.09	0.72	1.59	46.5	2.71	10.7	0.31	0.29	0.04	3.82	1.38

Mass concentrations of each trace metal are reported in ng/m^3^. Total PM_2.5_ mass concentrations are reported in µg/m^3^. Corresponding standard deviations can be found in the supplemental Table S4.

Whole-system uncertainties for the SPARTAN network were calculated in the same manner as previously reported by Weagle and coauthors^39^. The results for the uncertainties can be found in the supplemental Table S3, and individual plots in supplemental Figure S1.

Of interest is the anthropogenic contribution to such trace metals in general. Crustal enrichment factors (EFs) were used to distinguish naturally occurring crustal elements from those released by anthropogenic sources. We compared filter-extracted elemental concentrations *X* to background continental concentrations from Taylor and McLennan^40^, normalized by measured crustal Fe concentrations, which is predominantly from natural sources (e.g.^41^) as seen in Eq. (1).

1 $$E{F_{X,2.5}} = \frac{{{{\left[ {{\raise0.7ex\hbox{$X$} \!\mathord{\left/ {\vphantom {X {Fe}}}\right.\kern-\nulldelimiterspace} \!\lower0.7ex\hbox{${Fe}$}}} \right]}_{{\text{P}}{{\text{M}}_{2.5}}}}}}{{{{\left[ {{\raise0.7ex\hbox{$X$} \!\mathord{\left/ {\vphantom {X {Fe}}}\right.\kern-\nulldelimiterspace} \!\lower0.7ex\hbox{${Fe}$}}} \right]}_{{\text{Taylor}}}}}}$$

Although natural variability in the Fe fraction in soil will affect these results, the Fe fraction in soil tends to be quite consistent^42^. Anthropogenic sources of Fe imply that the resultant enhancement ratios will be a conservative indicator of anthropogenic contribution. Nonetheless, to ensure that conclusions are not affected by using Fe as the reference element due to extraction efficiency, a complementary analysis was performed by replacing site Fe concentrations with corresponding coarse PM concentrations, seen in Eq. (2). Coarse particulate matter over land has been established as predominantly stemming from suspended dust^43,44^ (with other potential sources including urban dust and organic material), making it a suitable measure of crustal source abundance to verify the validity of results using Fe. An estimate of the concentrations of the metal oxides present in coarse PM (estimated coarse PM = PM_c_^*^) was obtained by use of the soil reconstruction equation described by Malm et al.^43^ and shown in Eq. (3), in combination with continental concentrations from Taylor and McLennan.

2 $$E{F_{X,2.5}} = \frac{{{{\left[ {{\raise0.7ex\hbox{$X$} \!\mathord{\left/ {\vphantom {X {P{M_c}}}}\right.\kern-\nulldelimiterspace} \!\lower0.7ex\hbox{${P{M_c}}$}}} \right]}_{{\rm{P}}{{\rm{M}}_{2.5}}}}}}{{{{\left[ {{\raise0.7ex\hbox{$X$} \!\mathord{\left/ {\vphantom {X {PM_c{^*}}}}\right.\kern-\nulldelimiterspace} \!\lower0.7ex\hbox{${PM_c{^*}}$}}} \right]}_{{\rm{Taylor}}}}}}$$

3 $${\text{PM}}_{{\text{c}}}{^{*}} = { 2}.{2}0\left[ {{\text{Al}}} \right] + { 2}.{49}\left[ {{\text{Si}}} \right] + { 1}.{63}\left[ {{\text{Ca}}} \right] + { 2}.{42}\left[ {{\text{Fe}}} \right] + { 1}.{94}\left[ {{\text{Ti}}} \right]$$

**Figure 2:**
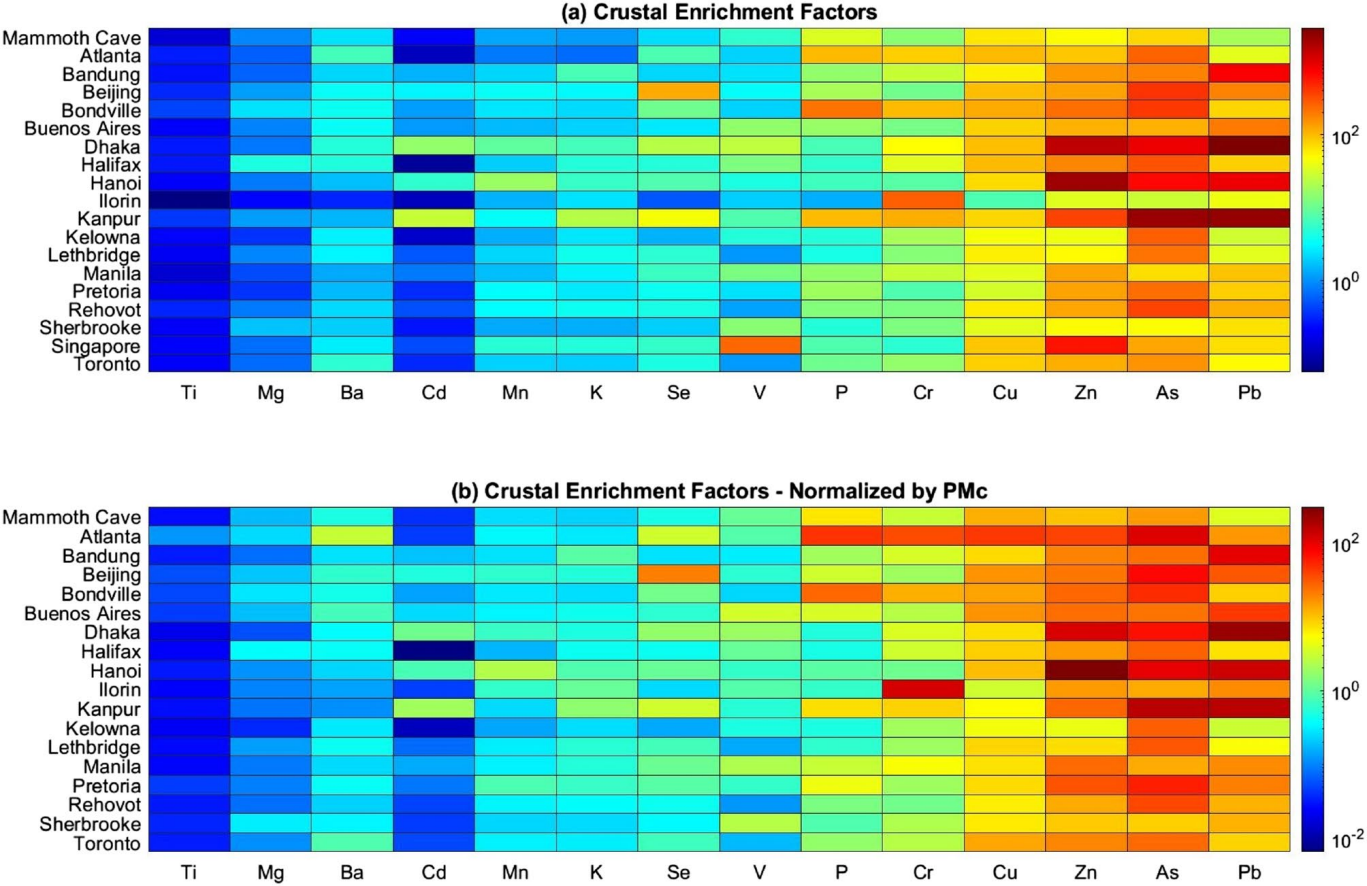
(**a**) Crustal enrichment factors (EF) for PM_2.5_ relative to crustal ratios of the given element and iron (Eq. 1), and (**b**) replacing measured iron with measured PM_c_ (Eq. 2). Elements are sorted by mean enrichment factor across all sites; sites are listed alphabetically. Singapore is not shown in the bottom plot due to unavailable PM_c_ data.

Enrichment factors can be considered in three subsets: EF < 10, elements which we attribute to sources that are primarily crustal^45–47^; EF between 10 and 100, elements with mixed anthropogenic and natural sources; and EF > 100, elements with largely anthropogenic sources. Figure 2 displays the EF elemental breakdown for all SPARTAN sites as well as the site-specific PM_c_-scaled EF results.

Similar results when normalizing by either Fe or PM_c_ provides confidence in the analysis. Enrichment factors vary by orders of magnitude depending on element and location. The highest levels of enrichment are found in anthropogenically-dominant elements such as Pb, As, and Zn, and generally found at sites with the highest levels of PM_2.5_. Of the four sites with the greatest PM_2.5_ concentrations, elemental EF values > 350 are found in Dhaka, Kanpur, and Hanoi for Pb, Zn, and As, and in Beijing for As. These enrichments highlight the effects of anthropogenic activities not only on total PM_2.5_ but on trace metal PM_2.5_ specifically, with potentially harmful levels of these heavy metals found in these high-PM cities.

**Figure 3:**
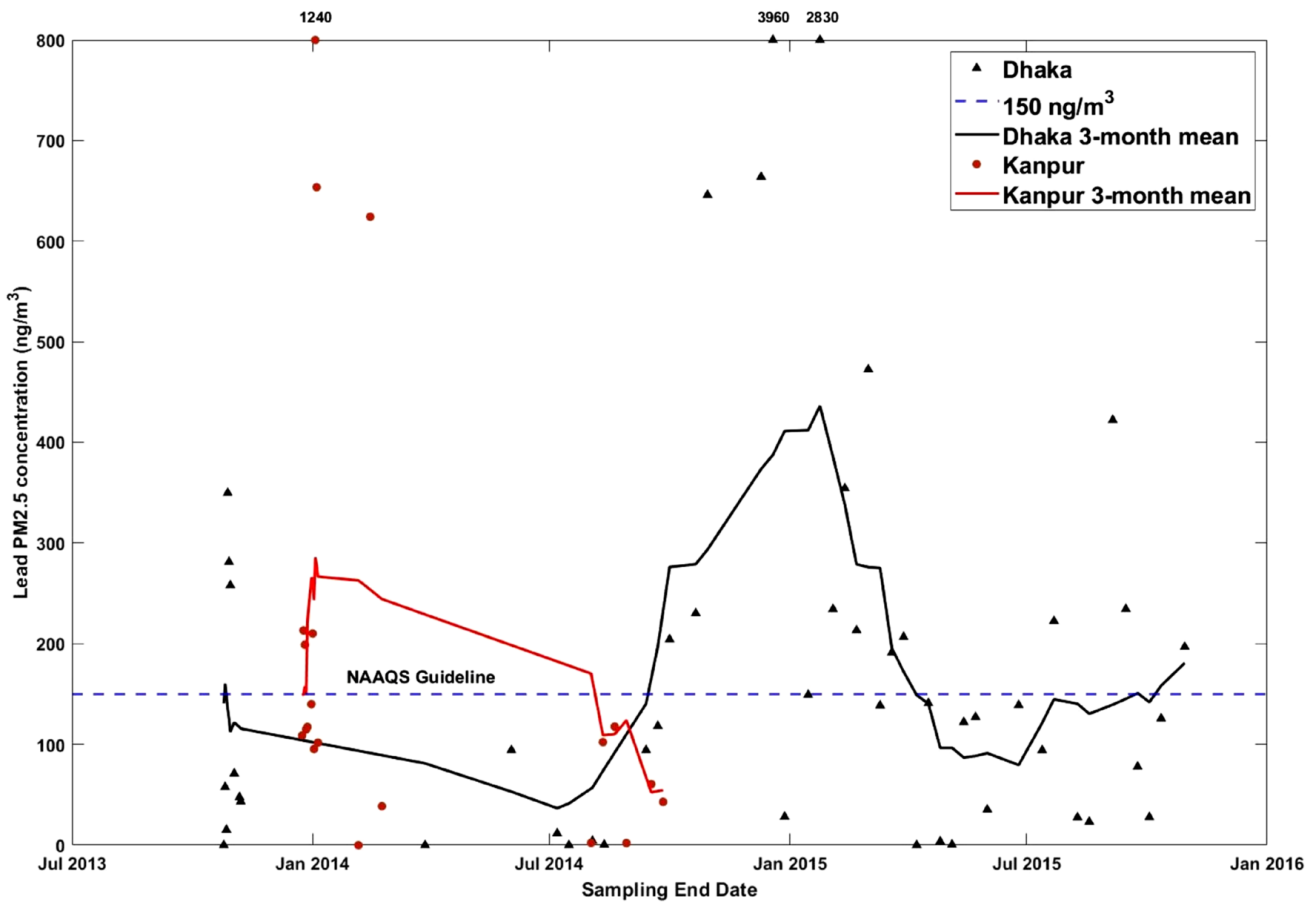
Concentrations of Pb in SPARTAN samples at Kanpur (red circles) and Dhaka (black triangles). Dotted blue line represents the NAAQS 3-month exposure guideline for lead concentrations (150 ng/m^3^). Samples are plotted by the final date of sampling, as sampling occurs over a time period of 9 days. Solid red (Kanpur) and black (Dhaka) lines represent 3-month rolling mean Pb concentrations.

Of the individual elements measured, two elements in particular demand further investigation: the carcinogenic metalloid As, and the heavy metal Pb. These elements can originate from multiple industrial sources, including smelting, waste incineration, and coal burning^12,14^. There are guidelines for the concentrations in air of both elements. For example, the US National Ambient Air Quality (NAAQS) exposure limit for Pb is a 3-month mean concentration of 150 ng/m^3^^48^, and multiple samples taken from the Kanpur and Dhaka sites exceeded these Pb concentrations—leading to 3-month mean concentrations over the US guideline for both sites. Figure 3 shows the lead PM_2.5_ concentrations measured in samples taken from the two sites, with the NAAQS guideline as a reference. This guideline refers to Total Suspended Particulate matter (TSP), a wider size range of particulates, so measured concentrations of lead PM_2.5_ are underestimating the TSP concentrations.

A comparison of the PM_2.5_ (Table 1) and PM_10_ (Table S7) values reveals that site-mean PM_10_ values are 41% higher at Kanpur and 74% higher at Dhaka than the PM_2.5_ values. Comparing PM_2.5_ at the two sites, Dhaka has both a higher mean Pb concentration (280 ng/m^3^) than Kanpur (209 ng/m^3^), and more samples measuring above the 150 ng/m^3^ guideline (38% vs. 30%). This is in contrast to their respective levels of total PM_2.5_, as mean PM_2.5_ concentrations from Kanpur samples are roughly double those in Dhaka. The Dhaka site exhibits some seasonality, with peak Pb values occurring around January 2015 and generally high values in winter months, when mixed layer depths are shallow. Similarly high Pb levels in PM_2.5_ have previously been reported in Dhaka^49,50^, and have been attributed to combustion of fossil fuels, battery industries, paints and varnishes, and lead-containing waste water.

**Figure 4:**
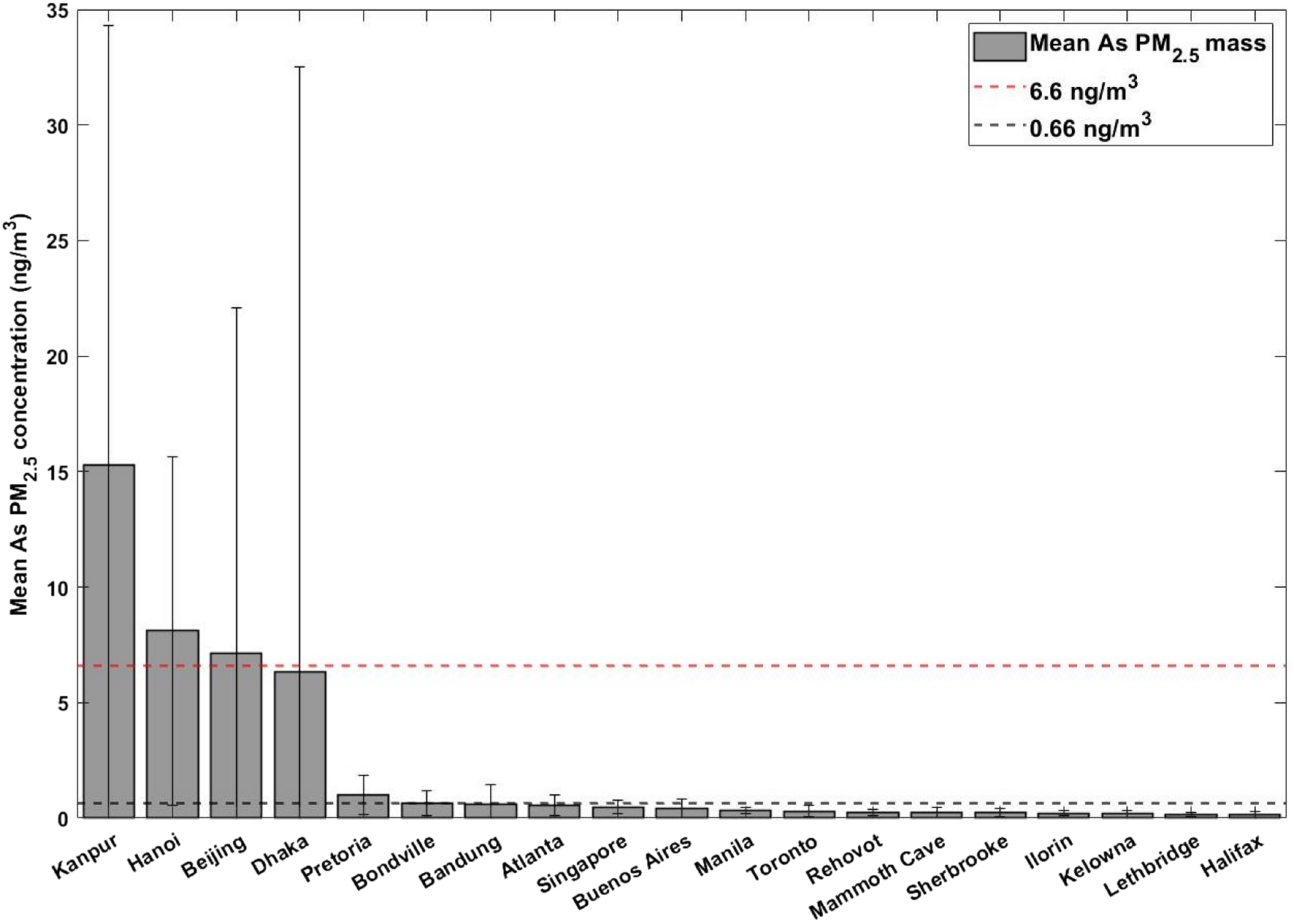
Concentrations of As in PM_2.5_ samples taken from SPARTAN sites, with standard deviation bars shown. Dotted red line represents 1:100,000 excess lifetime risk of cancer due to As exposure (6.6 ng/m^3^). Dotted black line represents 1:1,000,000 excess lifetime risk of cancer due to arsenic exposure (0.66 ng/m^3^).

The second element of interest in this study is arsenic, as the World Health Organization recommends no safe level of arsenic exposure due to its carcinogenic risk. An estimated lifetime excess risk is 1:1,000,000 at 0.66 ng/m^3^, or 1:100,000 at 6.6 ng/m^3^^51^^.^ As is associated with industrial activities such as smelting, burning of coal, and waste incineration^52–54^. Mean As concentrations for SPARTAN sites are shown in Fig. 4. The majority of SPARTAN sites fall under this level, but there are four sites with mean As concentrations near or above 6.6 ng/m^3^—Kanpur, Hanoi, Beijing, and Dhaka. The Beijing and Dhaka sites were sampled for an entire year facilitating direct comparison with the guideline. Although sampling at Kanpur and Hanoi was less than a year, concentrations at these sites were sufficiently high that the standard would be approached or exceeded even in the unlikely event that ambient concentrations were zero for the remainder of the year (yielding roughly 11.5 ng/m^3^ for Kanpur and 6.1 ng/m^3^ for Hanoi).

Kanpur has significantly higher As concentrations than any other SPARTAN site with 15.3 ng/m^3^. The mean As mass concentrations for Hanoi, Beijing, and Dhaka were determined to be 8.1 ng/m^3^, 7.1 ng/m^3^, and 6.3 ng/m^3^ respectively. These sites are also the four sites with the highest levels of PM_2.5_, so it is not unexpected that they have greater abundances of trace metal PM_2.5_ such as arsenic.

**Figure 5:**
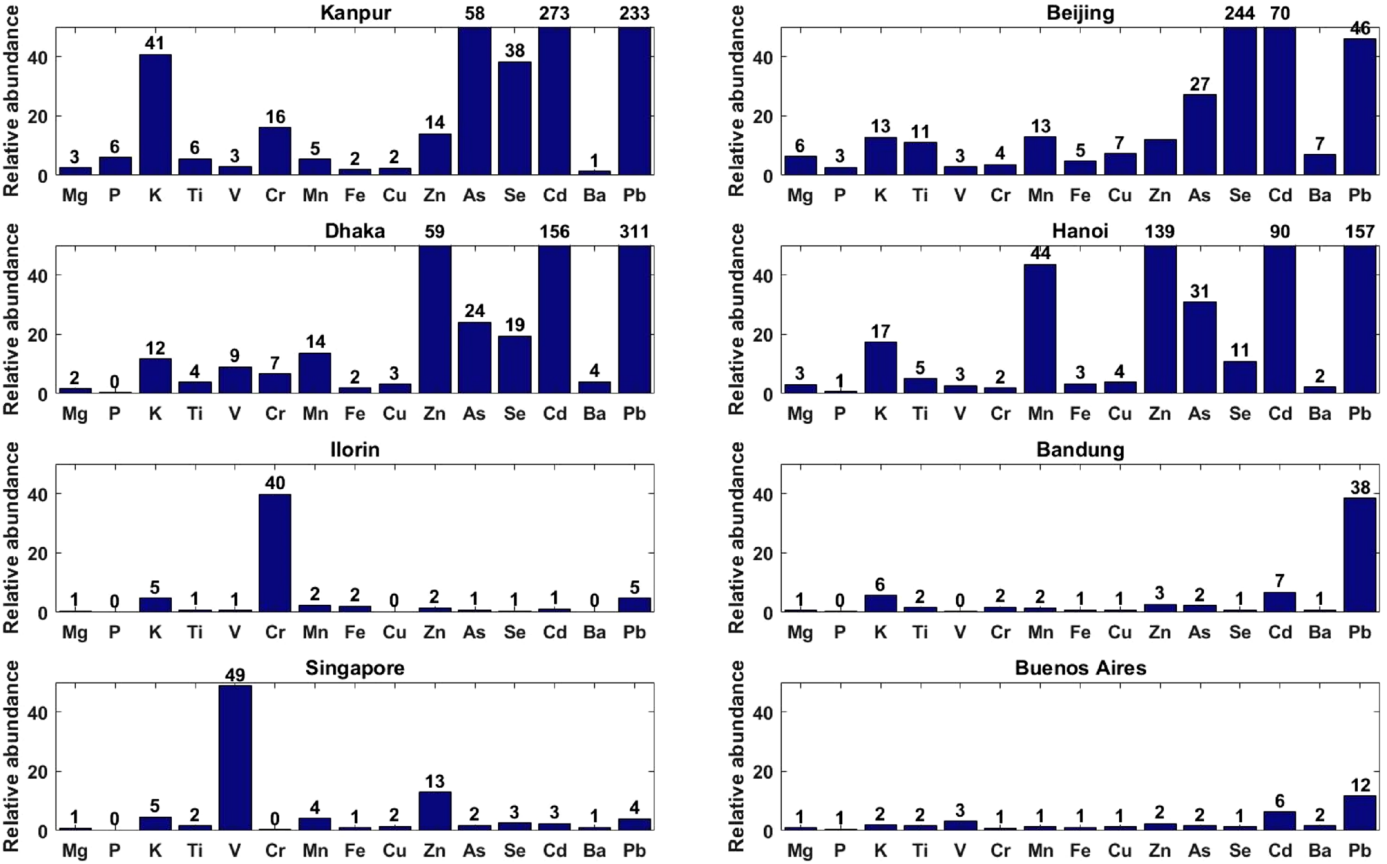
Relative abundances of trace metals in PM_2.5_ in representative SPARTAN sites with at least one element with RA of 10 or greater. Abundances are relative to the natural, low-trace metal PM_2.5_ Mammoth Cave site. Sites are sorted by total PM_2.5_ mass concentrations L-R, then top to bottom. Relative abundance values are unitless.

A complementary approach to understanding the levels of trace metals in fine PM at SPARTAN sites is to compare them not only to the crustal abundance, but to another site with relatively low levels of these trace metals. Figure 5 shows the relative abundance (RA) of PM_2.5_ trace metals at the eight SPARTAN sites with at least one elemental RA of 10 or greater, compared to a natural reference site, in this case the Mammoth Cave (M.C.) National Park site. The Mammoth Cave site has the second-lowest trace metal mass percentage of total PM_2.5_ mass. Low trace metals and the natural environment of the site make it an insightful reference point against which to compare the various types of SPARTAN sites. Relative abundances for trace metal PM_2.5_ are calculated using Eq. (4) and are unitless.

4 $$R{A}_{X}={\left[{\text{X}}\right]}_{\text{Site}}/{\left[{\text{X}}\right]}_{\text{M.C.}}$$

Sites with an elemental RA of 10 or greater are shown in the figure below to highlight particular areas and species of note. The full set of relative abundances can be found in the supplemental information (Table S5), as well as a similar calculation using the PM_2.5_-relative elemental concentrations instead of the absolute concentrations (supplemental Table S6). Generally, combustion-related elemental concentrations increase together, as do crustal components.

Particularly high RA values (12–311) of anthropogenic elements Zn, As, Pb, and Cd are found for Kanpur, Beijing, Dhaka, and Hanoi; these abundances are in alignment with the crustal enrichments seen for these heavy metals at these sites, and with the higher PM_2.5_ levels at these sites.

Examining the relative abundances on a site-specific basis reveals more local information—one notable instance being the Beijing site, which shows markedly elevated levels of selenium compared to any other site. Chinese coal has been found to be particularly rich in selenium^55^, which in concert with the large quantity of coal burned in China implies coal-burning as a major anthropogenic source of selenium PM_2.5_ in the region. Coal emissions in China have also been shown to contain high levels of As, Cd, and Pb^56^, all of which are significantly elevated by RA in the PM_2.5_ samples from Beijing.

There are a few isolated, notable RA values at the Bandung and Ilorin sites that can be linked to specific regional industries. Bandung has highly elevated Pb (RA of 38, EF > 700), even after the phasing out of leaded gasoline in 2006^57^. One likely contributor to the extremely high Pb levels is lead smelting, as Indonesia is one of the largest lead acid battery recyclers in Asia^58^. Rapidly growing numbers of lead smelters produce large amounts of Pb-enhanced emissions, the transport of which could explain the elevated lead levels seen at the Bandung site. The Ilorin site shows abundant levels of Cr (RA of 40, EF > 260); this may be associated with the prevalent tanning industry in the region, in which chromium compounds are used prominently. Analysis of effluents from Nigerian tanneries found high levels of Cr present^59^, and high levels of the metal in PM_2.5_ imply that some of this Cr is being converted to or released in particulate form.

Singapore has significant enrichment of V (49), which likely stems from the nearby burning of shipping fuel, with the Port of Singapore being one of the busiest ports in the world. Proximity to petroleum refineries is another likely contributor to these elevated V levels^60^, as the Singapore site is located approximately 10 km east-northeast of Jurong Island, an industrialized artificial island home to several refineries^61^. Significantly enhanced levels of V in Singapore can also be seen in the crustal EFs (EF > 240, the highest for V of any SPARTAN site), which further validates the key impact of nearby anthropogenic emissions. The influence of these and other nearby anthropogenic activities (e.g. vehicular traffic and metal production facilities) is also seen in enhanced levels of Zn, with an RA value of 13 and EF > 600. Unlike some of the other regions with high PM_2.5_ and significant anthropogenic activity, Singapore does not exhibit large relative abundances for other anthropogenically dominant elements such as Pb, As, and Cr. This is likely due to the near-total absence of coal-burning in the region, as Singapore relies heavily on natural gas for energy purposes^61^.

Two non-North American sites did not exhibit any RA values of 10 or higher: Manila and Rehovot. This merits attention as Manila in particular is a densely populated city yet shows no significantly higher levels of trace metals than our natural site in Mammoth Cave. One partial explanation is that Manila has relatively low PM_2.5_ mass concentrations among the non-North American sites (8th out of 11 sites). For Rehovot, the sampling site is the least densely populated non-North American site, which would align with the generally low amounts of trace metals observed at that location. Of the North American sites, Toronto, Bondville, Sherbrooke, Atlanta, Halifax, Kelowna, and Lethbridge all had RA values below 5 for all elements measured. This aligns well with expectations that these sites should generally have less PM pollution, especially for the Canadian MAPLE (Mortality Air Pollution Associations in Low Exposure Environments) sites designated as low-PM environments.

Although SPARTAN does not yet have sites in Europe, we surveyed prior measurements from the region to place our findings elsewhere in context. We find that at European background reference sites, heavy metal concentrations tended to be lower than at SPARTAN sites in densely populated regions^62,63^. These findings reinforce our conclusions about the enrichment of heavy metals compared with background reference sites.

## Conclusions and implications

Globally consistent measurements of airborne metal concentrations in PM_2.5_ are needed to assess and understand the distributions of ambient concentrations. Over 800 samples of fine particulate matter (PM_2.5_) from 19 unique sampling sites located across four continents were collected and analyzed using consistent protocols, with concentrations of 15 different trace metals reported. As the SPARTAN network continues to develop, these sampling protocols will continue to be improved to better report the chemical composition of trace metals in PM_2.5_. In general, it was found that several elements were enriched compared to their concentrations in the crust—in particular, elements such as Pb, As, and Zn that have anthropogenic sources were greatly enriched in sampled PM_2.5_. This enrichment was most notable in large, densely populated urban areas such as Beijing, Dhaka, Kanpur, and Hanoi, but was seen generally across many SPARTAN sites.

The enrichment of potentially harmful elements in fine particulate matter from anthropogenic sources is of global relevance to public health and warrants further attention. For example, we found in Dhaka and Kanpur that Pb concentrations exceeded the US National Ambient Air Quality 3-month guideline of 150 ng/m^3^, and that concentrations of the carcinogen As approached or exceeded the World Health Organization’s 1:100,000 excess lifetime risk level (6.6 ng/m^3^) in Kanpur, Hanoi, Beijing, and Dhaka. Emerging evidence indicates that anthropogenic fugitive, combustion, and industrial dust can comprise a significant fraction of PM_2.5_ in densely populated regions^64^. More generally, the high concentrations of several potentially harmful elements (e.g. Zn, Pb, Cd, Se, and As) at densely populated cities such as Beijing, Dhaka, Kanpur, and Hanoi motivate expanded measurements in other cities worldwide, especially in rapidly developing economies.

## Materials and methods

**Figure 6:**
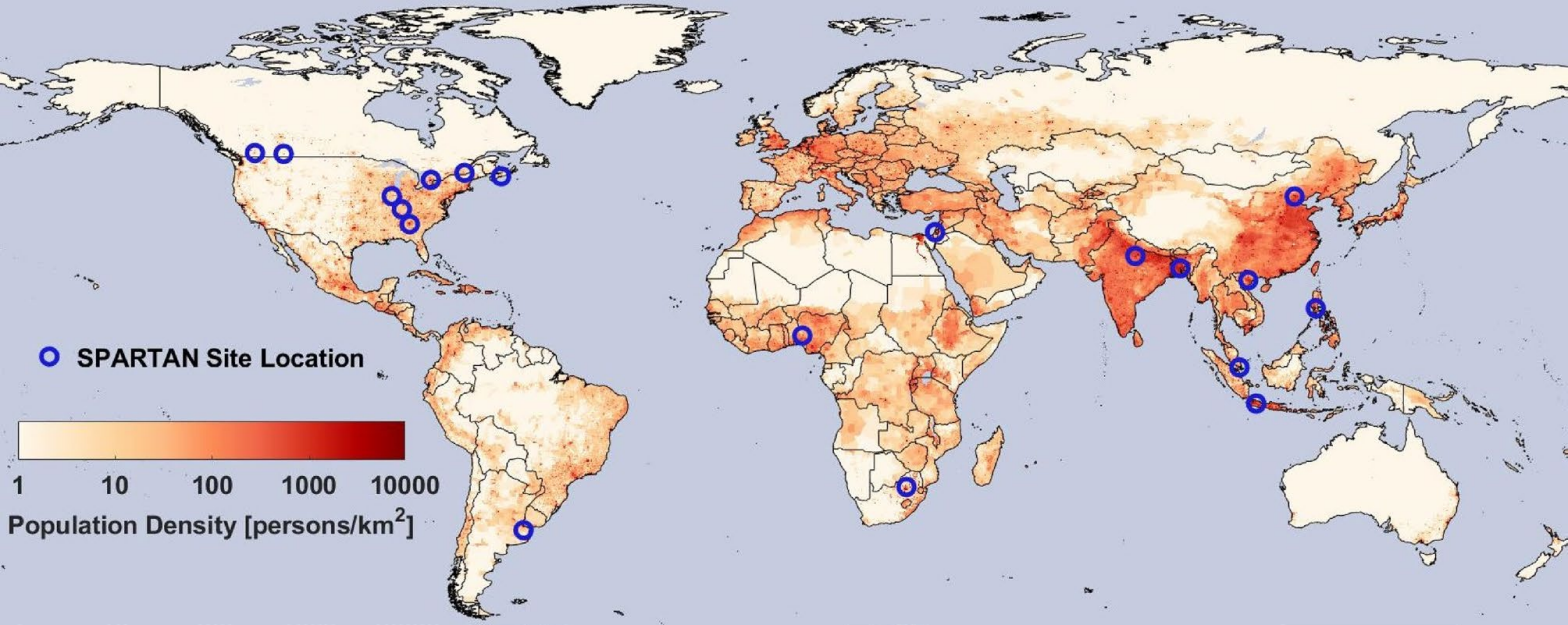
Open blue circles indicate the location of SPARTAN sampling sites used here, overlaid on a background color map of population density (NASA SEDAC GPW^65^, made using MATLAB R2019b—https://www.mathworks.com/products/matlab.html).

SPARTAN site-selection favors densely populated, globally dispersed regions that are underrepresented in terms of availability of representative and long-term air quality data. Local site-selection favors representative environments that avoid anomalous sources; low rooftops in urban environments are desirable to increase fetch, diminish local traffic influence, and offer instrument security. Locations of SPARTAN sites are shown in Fig. 6 and further site information (e.g. elevation, latitude, longitude, sampling period) is detailed in Table 2.

**Table 2:** SPARTAN site-by-site information.

City	Host Institute	Latitude	Longitude	Elevation (m)	Site type	Population density (/km^2^)^a^	Filters sampled	First sample end date	Last sample end date	Seasons sampled in^b^
Dhaka	University of Dhaka	23.728	90.398	34	Megacity ^c^	80,790	49	25-Oct-13	12-Oct-15	1,2,3,4
Bandung	Institute of Technology Bandung	− 6.888	107.610	826	Urban background	22,280	78	19-Jan-14	23-Dec-16	1,2,3,4
Hanoi	Vietnam Academy of Science	21.048	105.800	40	Urban background	21,430	12	09-Jun-15	09-Dec-17	1,3,4
Beijing	Tsinghua University	40.004	116.326	92	Megacity	18,300	171	03-Sep-13	05-Oct-17	1,2,3,4
Manila	Manila Observatory	14.635	121.078	63	Megacity	17,640	10	02-May-14	22-Oct-15	2,3,4
Pretoria	CSIR	− 25.757	28.280	1449	Urban background	13,400	4	27-Oct-15	29-May-16	2,4
Buenos Aires	CITEDEF	− 34.555	− 58.506	26	Megacity	9160	43	11-Oct-14	14-Oct-16	1,2,3,4
Singapore	National University of Singapore	1.298	103.780	30	Urban background	5460	41	14-Apr-16	14-Dec-17	1,2,3,4
Halifax	Dalhousie University	44.638	− 63.594	65	Urban background	5040	56	27-Aug-17	01-Apr-19	1,2,3,4
Toronto	Environment Canada	43.790	− 79.470	186	Urban background	3800	61	13-Jul-17	10-Mar-19	1,2,3,4
Kanpur	IIT Kanpur	26.513	80.232	123	Urban background	3250	21	23-Dec-13	26-Sep-14	1,2,3,4
Ilorin	University of Ilorin	8.484	4.675	400	Urban background	1620	17	29-Jun-14	23-Apr-19	1,2,3,4
Rehovot	Weizmann Institute	31.907	34.811	73	Urban background	1440	76	22-Feb-15	03-Oct-18	1,2,3,4
Sherbrooke	Université de Sherbrooke	45.380	− 71.931	251	Urban background	1190	52	06-Jul-17	20-Mar-19	1,2,3,4
Lethbridge	University of Lethbridge	49.682	− 112.869	904	Urban background	590	30	03-Sep-17	23-Jan-19	1,3,4
Atlanta	Emory University	33.688	− 84.290	250	Suburban	540	21	27-Jan-14	22-Apr-14	1,2
Kelowna	Environment Canada	49.941	− 119.400	456	Suburban	61	17	10-Nov-17	06-Mar-19	1,2,4
Mammoth Cave	Mammoth Cave National Park	37.132	− 86.148	235	Natural	13	21	09-Jun-14	12-Aug-14	3
Bondville	University of Illinois	40.053	− 88.372	200	Rural	2	21	20-Aug-15	14-Apr-16	1,2,3,4

Sites are sorted from highest population density to lowest.

^a^Population density is reported for a 1 km radius based on NASA’s Gridded Population of the World^65^.

^b^For concision, seasons are labelled as such: 1—Dec–Feb, 2—Mar–May, 3—Jun–Aug, 4—Sept–Nov.

^c^Megacities are defined as having 10 million or more inhabitants^66^.

As SPARTAN site selection prioritizes under-sampled locations, some regions that are well-represented in terms of air quality data have not established a SPARTAN site to date. Further expansion of the network is planned for the future, in order to cover regions not sampled by SPARTAN, as well as add more sites in underrepresented continents.

Sampling through SPARTAN has also occurred at several North American sites through various projects. Three pilot sites in the United States sampled in 2014–2016, and five sites located across Canada sampled over 2017–2019 as part of the MAPLE project^67^. Overall filter sampling occurred over an average of 20 months across sites (range of 2 to 50 months). Over 800 PM_2.5_ filters have been analyzed as of October 2019 and were included in the data set used for this study. While some sites may have lower numbers of samples, the SPARTAN sampling protocol (detailed below) was designed to better capture long-term averages in sampled PM, so even relatively few samples could be representative for the season in which they were sampled (detailed in Table 2). Ongoing measurements are expected to increase seasonal representativeness over time.

An overview of SPARTAN is provided by Snider et al.^37,68^, in which the basis of the sampling procedure, filter analysis, and measurement ranges are presented. As the SPARTAN project developed, some aspects of the process were updated. To briefly summarize the original procedure, PM_c_ (coarse PM) and PM_2.5_ filter masses were collected on a two-stage stacked filter unit inside rooftop-mounted AirPhoton SS4i automated air samplers over 9-day periods. A removable filter cartridge protected seven sequentially active pairs of coarse Nuclepore and fine Teflon filters, plus a pair of travelling blanks. Beginning in late 2017, sampling stations at SPARTAN were upgraded to the AirPhoton SS5 models, which use a cyclone inlet to separate particles by varying flow rates through the station (5 and 1.5 L/min, respectively, for PM_2.5_ and PM_10_ size-cuts). This allowed for elimination of the Nuclepore filters and the introduction of filter cartridges with eight total stretched Teflon filters that included six sampling PM_2.5_, one sampling PM_10_, and one travelling blank. These cartridges were pre-assembled in the SPARTAN central laboratories at Dalhousie University, and shipped to sites for installation by site operators.

Once the filter cartridges are installed, the sampling stations run autonomously for the cartridge duration. For international sites, each of the PM_2.5_ filters is sampled for rotating 3-h spans over 9 days, totaling 24 h of sampling. Measuring across the entire diurnal cycle over a 9-day period, rather than sampling for a consecutive 24-h period every 9 days, helps to better capture long-term averages^37^. The PM_10_ filter was sampled for a 30-min period after each 3-h PM_2.5_ sample for the entire 54-day sampling period, providing a 54-day average PM_10_ concentration over a total of 24 h. For sites that are part of the MAPLE project, each filter was sampled for a total of 48 h (6-h periods for the PM_2.5_ filters and 1-h periods for the PM_10_ filters) to ensure a quantifiable amount was deposited in these low-PM environments. Once sampling was complete, filter cartridges were removed from the sampling station, sealed, and returned to the central laboratory at Dalhousie University for chemical and physical analysis of the filters. Filters were analyzed to determine PM_2.5_ or PM_10_ mass (gravimetric), water-soluble ions (ion chromatography with conductivity detection), black carbon (absorbance, determined through smoke-stain refractometry), and trace metals (inductively-coupled plasma mass spectrometry).

The focus of this study is trace metal content, quantified here via inductively coupled plasma mass spectrometry (ICP-MS, Thermo Scientific X-Series 2). For each filter a small quantity of isopropyl alcohol (previously 30 µL, currently 10 µL) was added, then the filter was extracted (97 °C for 2 h) with 5% trace metal grade nitric acid solution in a process similar to Fang et al.^20^ and Herner et al.^69^. Filters were boiled in the acidic solution and the liquid extract submitted for quantitative analysis via ICP-MS, using 25–500 ppb (μg/L) trace metals standards and three reference elements for atomic mass (^45^Sc, ^115^In, and ^159^Tb) for each analysis. Measured concentrations from the field blank filters for each filter cartridge were subtracted from the seven corresponding samples of each cartridge to account for variable trace metal baselines. Consistent ICP-MS analysis in the central Dalhousie laboratory facilitated consistency of results across sites. It is established that nitric acid extraction efficiencies for some crustal elements such as Fe can be as low as 50%^70^ as confirmed by our internal tests, and as such, measurement methods are being re-evaluated for future SPARTAN use. More recent analyses include the use of HCl to improve extraction efficiencies. In addition, filters will also be analyzed with XRF at Washington University. For now, this initial analysis offers perspective on this emerging dataset, with observed variability that far exceeds the factor of 2 uncertainty associated with some extraction efficiencies.

In order to compare SPARTAN trace metal measurements with independent concurrent measurements, a joint sampling campaign was conducted in the US in concert with the IMPROVE (Interagency Monitoring of Protected Visual Environments) network that has been previously described^68^ and is summarized in the supplemental information (section S2).

Whole system uncertainties for the SPARTAN network are estimated through use of collocated filter sampling stations. The process is described in previous work^39^ but briefly, three sites in typically low (Halifax, Canada), moderate (Toronto, Canada), and high (Beijing, China) PM environments performed collocated sampling over 3 week periods. Over this period, each station recorded 24-h samples (48-h in Halifax to ensure adequate loading) which were then analyzed to evaluate uncertainties across the network as further described in the supplemental material (section S3).

## Supplementary Information

Supplementary Information.
