# Supplementary material for: Large global variations in measured airborne metal concentrations driven by anthropogenic sources
Source: Sci Rep. 2020 Dec 11;10:21817. doi: 10.1038/s41598-020-78789-y (PMC7733447; doi:10.1038/s41598-020-78789-y)
Supplement: Supplementary file 1 — Supplementary Information. [file 41598_2020_78789_MOESM1_ESM.docx]

Supplemental Information for **“Large Global Variations in Measured Airborne Metal Concentrations Driven by Anthropogenic Sources”**

Jacob McNeill^[[1]](#footnote-1),^^[[2]](#footnote-2)^, Graydon Snider^[[3]](#footnote-3)^, Crystal L. Weagle^3,2^, Brenna Walsh^3,2^, Paul Bissonnette^3^, Emily Stone^3^, Ihab Abboud^[[4]](#footnote-4)^, Clement Akoshile^[[5]](#footnote-5)^, Nguyen Xuan Anh^[[6]](#footnote-6)^, Rajasekhar Balasubramanian^[[7]](#footnote-7)^, Jeffrey R. Brook^[[8]](#footnote-8)^, Craig Coburn^[[9]](#footnote-9)^, Aaron Cohen^[[10]](#footnote-10)^, Jinlu Dong^[[11]](#footnote-11)^, Graham Gagnon^[[12]](#footnote-12)^, Rebecca M. Garland^[[13]](#footnote-13),^^[[14]](#footnote-14),^^[[15]](#footnote-15)^, Kebin He^[[16]](#footnote-16)^, Brent N. Holben^[[17]](#footnote-17)^, Ralph Kahn^17^, Jong Sung Kim^[[18]](#footnote-18)^, Nofel Lagrosas^[[19]](#footnote-19)^, Puji Lestari^[[20]](#footnote-20)^, Yang Liu^[[21]](#footnote-21)^, Farah Jeba^[[22]](#footnote-22)^, Khaled Shaifullah Joy^22^, J. Vanderlei Martins^[[23]](#footnote-23)^, Amit Misra^[[24]](#footnote-24)^, Leslie K. Norford^[[25]](#footnote-25)^, Eduardo J. Quel^[[26]](#footnote-26)^, Abdus Salam^22^, Bret Schichtel^[[27]](#footnote-27)^, S.N. Tripathi^24^, Chien Wang^[[28]](#footnote-28)^, Qiang Zhang^16^, Michael Brauer^[[29]](#footnote-29)^, Mark D. Gibson^[[30]](#footnote-30),^^[[31]](#footnote-31)^, Yinon Rudich^[[32]](#footnote-32)^, Randall V. Martin*^2,1,3^

**S1 Overview of individual elements**

Summary information draws on Table 1. Species within a particular size fraction are referred to as X_PM2.5_ or X_PMc_.

**S.1.1** *Potassium (K)* is strongly associated with wood smoke ^3^ and also derives from natural crustal sources ^4^. We find the highest concentrations of K_PM2.5_ in Kanpur (3050 ng/m^3^), also where the highest organic (residual matter) concentration across SPARTAN sites was found ^5^.

**S.1.2** *Magnesium (Mg)* is coarse-mode dominant, and chiefly derives from mineral dust ^6,7^, as well as marine sources ^8^.

**S.1.3** *Phosphorus (P)* is generally crustal in nature ^9^, with roughly equal occurrence in fine and coarse modes, and can also arise from fertilizer. Of SPARTAN sites, Kanpur was found to have the highest concentration of P_PM2.5_, with 340ng/m^3^.

**S.1.4** *Titanium (Ti)* is mainly crustal in nature ^7^ but with a concentration less than 5% compared to Fe, Al, and Mg ^9^. We find Ti is primarily in the coarse mode (79%). The PM_2.5_ component may originate in part from coal combustion ^10^. We find maximum Ti_PM2.5_ concentrations in Beijing (11 ng/m^3^), where both crustal sources and coal combustions are enhanced.

**S.1.5** *Vanadium (V)* originates from fuel oil combustion ^10,11^, either from land-based vehicles or ships ^12,13^. We find V mass is slightly coarse-mode dominant (62%). Vanadium is a respiratory irritant, derived from its production of reactive oxygen species ^14^. We find the highest mean concentration of V_PM2.5_ in Singapore (38 ng/m^3^), which has an abundance of port activities and oil refining facilities.

**S.1.6** *Chromium (Cr)* is derived from combustion processes (e.g. coal) and steel dust ^15^ and is an irritant to the lungs ^16^. We find the maximum concentration of Cr_PM2.5_ in Ilorin (48 ng/m^3^), which may be due the prevalence of tanneries in the region ^17^ where large quantities of chromium compounds are used.

**S.1.7** *Manganese (Mn*) is mainly crustal in nature ^9^ but can be enhanced slightly due to vehicles ^18^ and from coal combustion, as well as industrial sources ^10^. We find that Mn is primarily in the coarse mode (68%) with a maximum PM_2.5_ concentration of 80 ng/m^3^ in Hanoi.

**S.1.8** *Iron (Fe)* is predominantly crustal in origin, coarse-mode dominant (72%), and chiefly derives from natural dust (Malm et al., 1994). Fine particulate Fe may also come from vehicles ^19^ or some industrial sources ^20^. We find maximum Fe_PM2.5_ concentrations of 390 ng/m^3^ in Beijing. Iron is used to reference enrichment of other elements given its mainly natural origin.

**S.1.9** *Copper (Cu)* is coarse-mode dominant and associated with vehicular traffic, brake wear, and zinc emissions ^21^. Cu in fine particulates is a source of lung inflammation and reactive oxygen species ^16,22^. The highest fine mode value is found in Beijing (27 ng/m^3^).

**S.1.10** *Zinc (Zn)* is fine-mode dominant and frequently attributed to tire wear ^13,23–25^ but can also be associated with other industrial activities such as non-ferrous metal production ^10,12,13,26,27^. Zinc is considered a source of oxidative stress ^16^. We find the highest fine mode values in Hanoi (1180 ng/m^3^).

**S.1.11** *Arsenic (As)* is associated with various industrial activities such as smelting, waste incineration, and coal ^28–30^. Higher doses of this water-soluble metal are toxic to humans ^31^. Arsenic is also a carcinogen ^32^. We find As generally in fine-mode aerosols (56%), with the highest PM_2.5_ concentrations found in Kanpur (15 ng/m^3^), possibly due to smelting ^33^, .

**S.1.12** *Selenium (Se)* has a significant source from coal ^10^. We find Se primarily in PM_2.5_ (64%), consistent with previous work ^34^. Se_PM2.5_ concentrations are highest in Beijing (67 ng/m^3^), where intense coal burning occurs regionally.

**S.1.13** *Cadmium (Cd*) is associated with coal and stationary combustion ^29^ as well as non-ferrous metal production ^10^. It may also be associated with vehicular activity ^26,27^. High concentrations can induce damage to kidneys, as well as the respiratory system ^35^. We find Cd fine-mode dominant (59%) with the highest PM_2.5_ concentration recorded in Kanpur (13 ng/m^3^).

**S.1.14** *Barium (Ba)* derives in part from natural dust sources ^36^, from motor vehicle emissions such as tire and brake wear ^12,13,25,37–39^, and from coal combustion ^40^. We find the highest PM_2.5_ concentrations in Beijing (22 ng/m^3^).

**S.1.15** *Lead (Pb)*: Lead comes from a mixture of leaded gasoline, coal, diesel engines, waste incineration, and other combustion ^27,33,41–43^. Increased levels of Pb intake can lead to low birth weight and impaired cognitive function in children ^44^. We find Pb frequently resides in fine-mode aerosols (58%) with the highest PM_2.5_ concentrations in Dhaka (280 ng m^-3^) and Kanpur (200 ng m^-3^), where concentrations exceed the US National Ambient Air Quality exposure limit of 150 ng m^-3^ ^45^, as well as Hanoi (140 ng m^-3^).

Table S1: SPARTAN network-wide statistics of fine fractions, element limits of detection, and samples above LOD for individual elements quantified by ICP-MS.

| **Element** | **Fine fraction^a^** | **LOD (ppb)^b^** | **Samples above LOD (%)** |
| --- | --- | --- | --- |
| Mg | 0.21 | 10 | 61 |
| Al | 0.23 | 4 | 78 |
| Li | 0.35 | 0.4 | 7 |
| P | 0.47 | 10 | 30 |
| Ti | 0.21 | 0.5 | 76 |
| V | 0.38 | 0.4 | 39 |
| Cr | 0.31 | 0.4 | 46 |
| Mn | 0.32 | 0.8 | 79 |
| Fe | 0.28 | 7 | 90 |
| Co | 0.18 | 0.4 | 2 |
| Ni | 0.48 | 0.4 | 50 |
| Cu | 0.37 | 0.7 | 75 |
| Zn | 0.48 | 0.6 | 79 |
| As | 0.56 | 0.4 | 47 |
| Se | 0.64 | 1 | 12 |
| Ag | 0.25 | 0.4 | 9 |
| Cd | 0.59 | 0.4 | 12 |
| Ba | 0.31 | 0.5 | 76 |
| Ce | 0.17 | 0.4 | 2 |
| Pb | 0.58 | 0.4 | 90 |

^a^Fine fraction for element *x* is reported as the geometric mean of the ${\bar{x_{2.5}}}/{\bar{x_{10}}}$ fractions for all SPARTAN sites.

^b^Limits of detection (LOD) are determined for the ICP-MS instrument for each individual element.

**S2** **Comparison of SPARTAN with IMPROVE**

In order to compare SPARTAN trace metal measurements with independent concurrent measurements, a joint sampling campaign was conducted in the US in concert with the IMPROVE (Interagency Monitoring of Protected Visual Environments) network. Table S2 details this comparison, where rows show results from the three sampling campaigns versus standardized instrumentation and sampling techniques from Atlanta ^46^, Bondville ^47^ and Mammoth Cave ^47^. Excellent agreement is apparent at both Atlanta (*m* = 1.05 ± 0.16, *r* = 0.95) and Mammoth Cave (*m* = 1.01 ± 0.17, *r =* 0.94). Trace metals in Bondville are closer to SPARTAN detection limits, which may explain the slightly larger difference in concentrations there.

Table S2: Correlations and slopes (axis-free regression) of SPARTAN PM_2.5_ versus collocated studies conducted by EPA’s IMPROVE network. Elements included are those consistently above SPARTAN limits of detection.

| Site | Size fraction | Number of species  co-measured | Collocated sampling,  log-log plot  Slope  ± 1σ-error *r* | | Reference Study |
| --- | --- | --- | --- | --- | --- |
| Atlanta | PM_2.5_ | 22 | 1.05 ± 0.16 | 0.95 | ^48^ |
| Bondville | PM_2.5_ | 20 | 0.90 ± 0.21 | 0.88 | ^47^ |
| Mammoth Cave | PM_2.5_ | 22 | 1.01 ± 0.17 | 0.94 | ^47^ |
| 3 USA  sites merged | PM_2.5_ | 23 | 0.99 ± 0.12 | 0.92 | EPA + IMPROVE |

**S3 Whole system uncertainties**

Whole system uncertainties for the SPARTAN network are estimated through use of collocated filter sampling stations. The process is described in previous work by the SPARTAN team ^49^ but in summary, three sites in typically low (Halifax, Canada), moderate (Toronto, Canada), and high (Beijing, China) PM environments performed collocated sampling over three week periods. Over this period, each station recorded 24-hour samples (48-hour in Halifax to ensure adequate loading) which were then analyzed as per SPARTAN protocol. This allows for a comprehensive evaluation of uncertainties across the network, as the sampling and analysis processes are duplicated for each sample in the collocated pair, but this approach may not account for systematic errors in analysis techniques. The uncertainty calculation is based on the US Code of Federal Regulations, Part 58 (Ambient Air Quality Surveillance), Appendix A, Section 4.2. For each collocated data pair, the relative percent difference, di, is calculated using equation S1:

|  | $d_{i}=\frac{X_{i}-Y_{i}}{\left( X_{i}+Y_{i} \right)^{2}}*100$ | S1 |
| --- | --- | --- |

where Xi and Yi are the species concentrations from the two sampling stations. The coefficient of variation upper bound is then calculated using equation S2:

|  | $\text{CV}\left( \text{upper bound} \right)=\sqrt{\frac{n*\sum_{i=1}^{n} d_{i}^{2}-\left( \sum_{i=1}^{n} d_{i} \right)^{2}}{2n(n+1)}}*\sqrt{\frac{n-1}{\chi_{0.1,n-1}^{2}}}$ | S2 |
| --- | --- | --- |

where n is the number of data pairs, and ${}_{0.1,n-1}^{2}$is the upper 10^th^ percentile of a chi-squared distribution with n – 1 degrees of freedom. The factor of 2 in the denominator adjusts for the error in d_i_ from two measurements.

Whole system uncertainties for the trace metals analyzed during this collocation are shown in Table S3, with both site-specific values as well as values for the whole SPARTAN network. Network-average values were lowest for PM_2.5_ (14.7%), Pb (15.4%), P (16.9%), and Fe (16.9%). Plots for individual components are shown in Figure S1.

Table S3: Uncertainties (%) as calculated through equation S2 for PM­_2.5_ and trace metals quantified during collocated sampling.

| **Location** | **# of filters** | **PM2.5** | **As** | **Co** | **Cr** | **Cu** | **Fe** | **Li** | **Mg** | **Mn** | **P** | **Pb** | **Ti** | **V** | **Zn** |
| --- | --- | --- | --- | --- | --- | --- | --- | --- | --- | --- | --- | --- | --- | --- | --- |
| Halifax | 18 | 9.8 | 17 | 30.1 | 19.4 | 32.7 | 10.6 | 23.2 | 33.5 | 33.1 | 9.1 | 8.7 | 12.3 | 22 | 55.9 |
| Toronto | 18 | 14.6 | 15.2 | 36.8 | 41.0 | 26.1 | 13.4 | 23.9 | 37.5 | 17.1 | 20.5 | 8.8 | 17.5 | 10.7 | 30.5 |
| Beijing | 14 | 16.5 | 24.1 | 24.9 | 15.7 | 19.3 | 16.9 | 28.9 | 14 | 21.1 | 13.9 | 21.5 | 20.7 | 31 | 18.2 |
| SPARTAN | 50 | 14.7 | 20.4 | 34.7 | 31.5 | 28.3 | 16.9 | 28.2 | 32.7 | 27.2 | 16.9 | 15.4 | 18.9 | 23.4 | 39 |


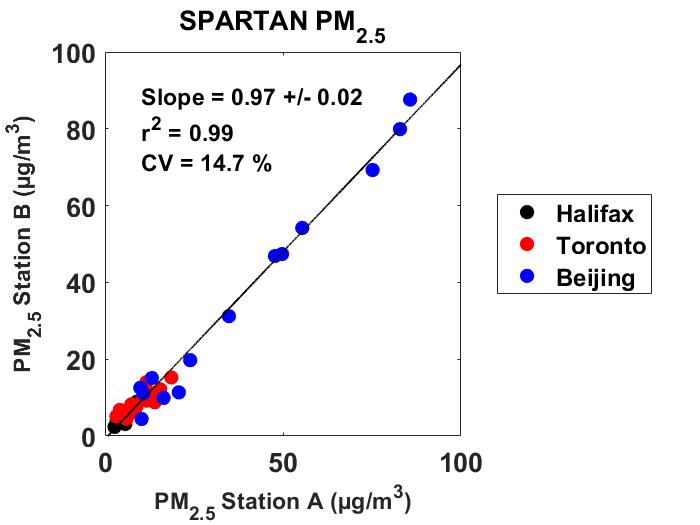

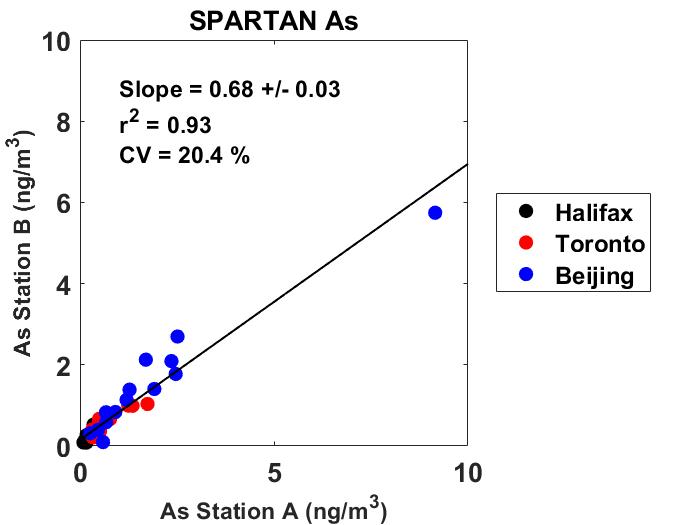

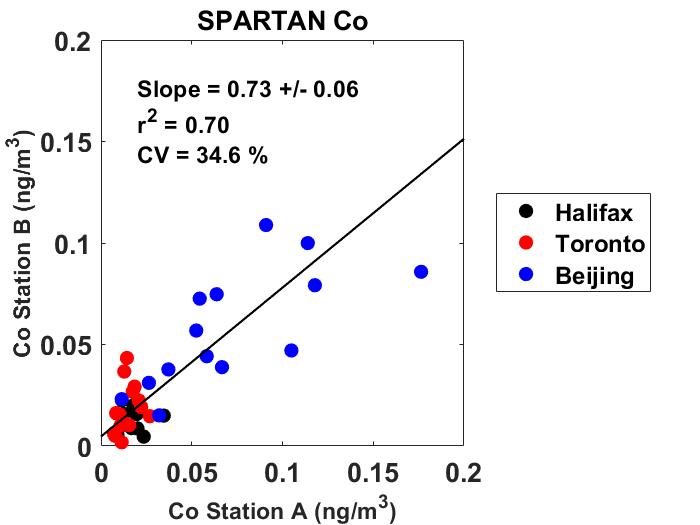

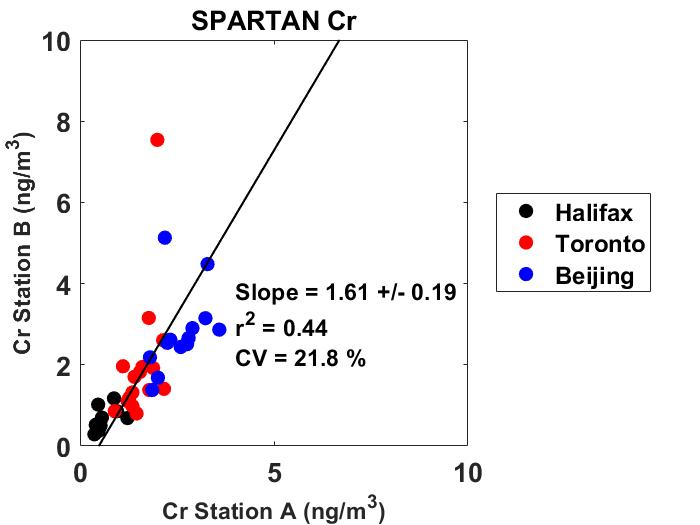

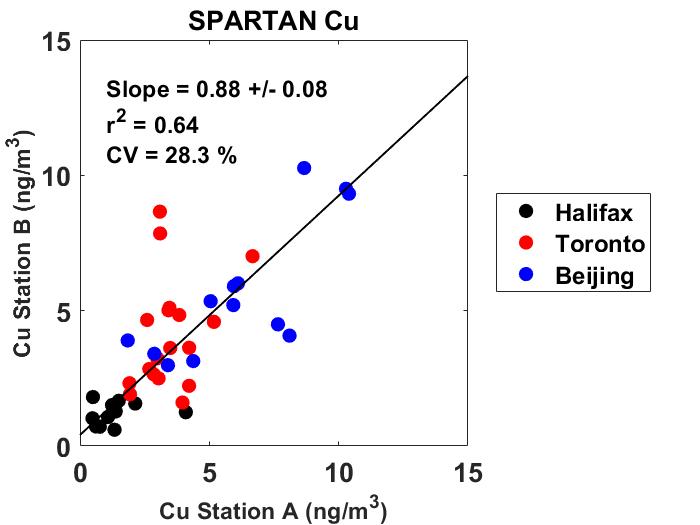

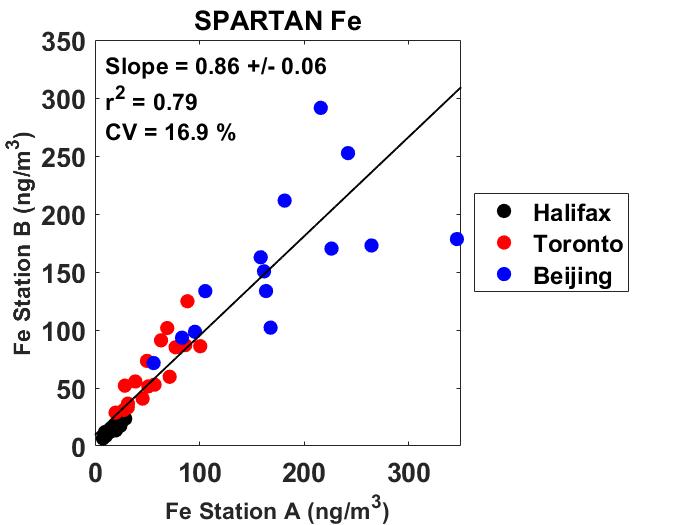

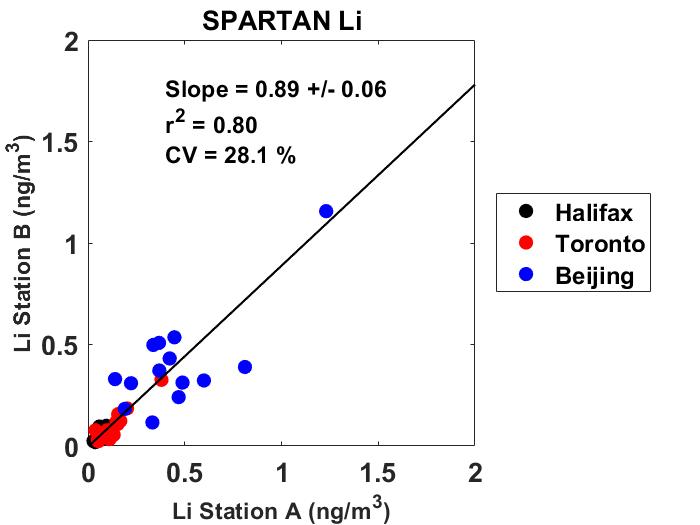

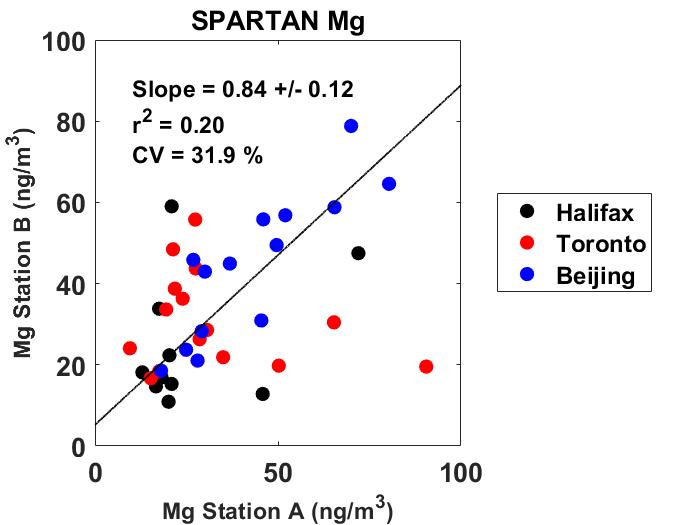

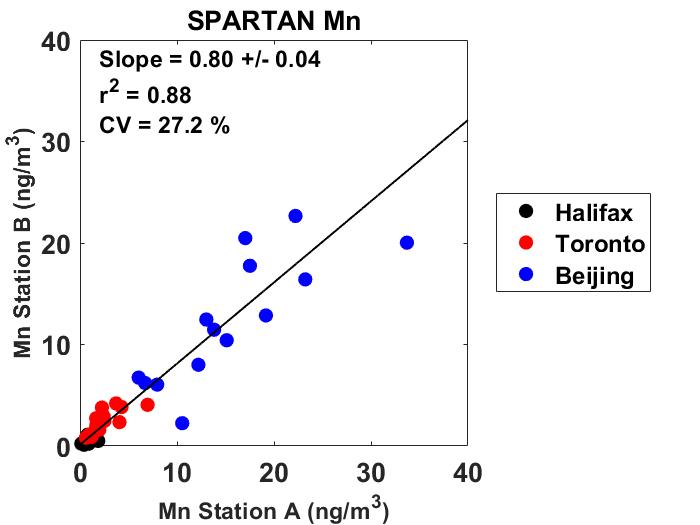

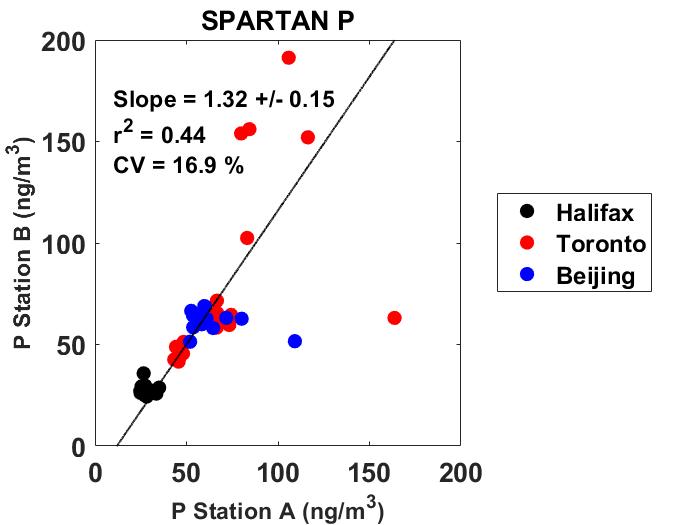

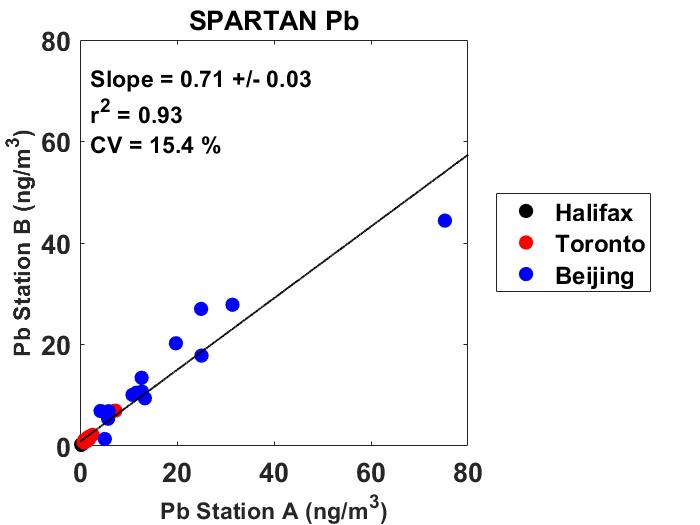

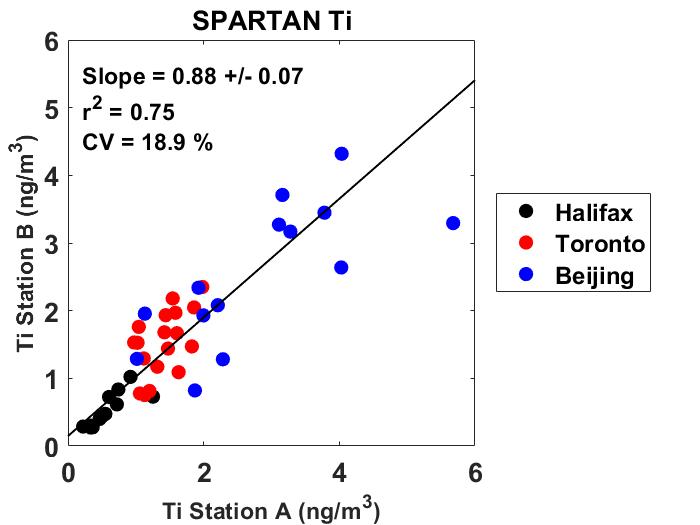

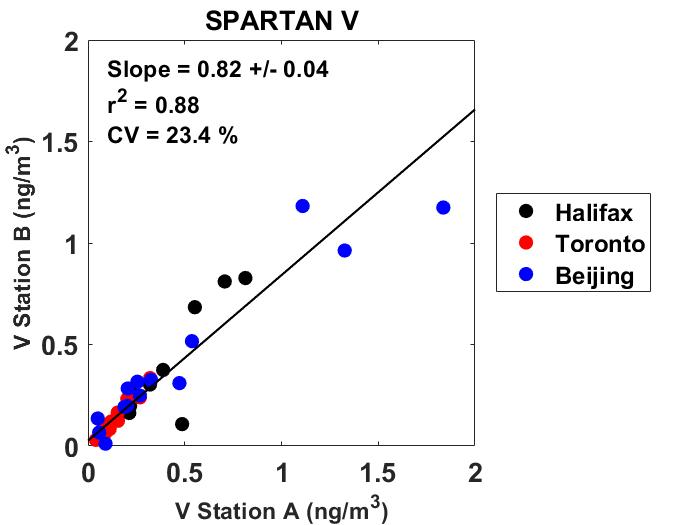

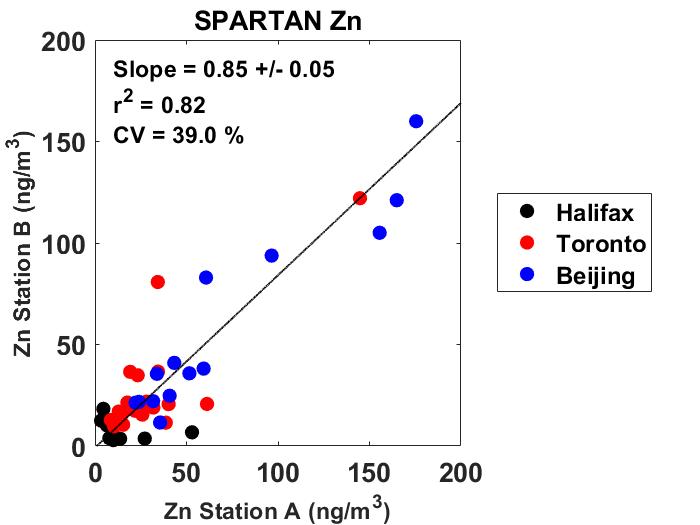


Figure S1: Individual plots of measured components at collocated sites in Halifax, Toronto, and Beijing used to estimate SPARTAN network uncertainties. CV shown in the plots represents the coefficient of upper bound calculated using Equation S2.

Table S4: Full elemental breakdown of standard deviations (population) for trace metals in PM_2.5_ for SPARTAN sites. Mass standard deviations are reported in ng/m^3^. PM_2.5_ standard deviations are reported in µg/m^3^.

|  | **PM_2.5_** | **K** | **Mg** | **P** | **Ti** | **V** | **Cr** | **Mn** | **Fe** | **Cu** | **Zn** | **As** | **Se** | **Cd** | **Ba** | **Pb** |
| --- | --- | --- | --- | --- | --- | --- | --- | --- | --- | --- | --- | --- | --- | --- | --- | --- |
| **Mammoth Cave** | 13.5 | 72.0 | 35.1 | 17.24 | 1.21 | 0.60 | 1.39 | 2.61 | 109.8 | 4.22 | 6.2 | 0.13 | 0.17 | 0.05 | 2.16 | 0.37 |
| **Atlanta** | 8.5 | 21.7 | 5.2 | 60.12 | 0.83 | 0.11 | 5.09 | 0.50 | 37.3 | 2.36 | 6.6 | 0.45 | 0.30 | 0.02 | 3.64 | 0.69 |
| **Buenos Aires** | 9.2 | 57.0 | 16.3 | 34.25 | 1.08 | 2.57 | 2.14 | 1.44 | 57.1 | 4.15 | 22.8 | 0.39 | 0.31 | 0.68 | 8.99 | 24.76 |
| **Bandung** | 28.5 | 205.7 | 17.7 | 46.37 | 0.97 | 0.19 | 5.79 | 2.35 | 90.3 | 6.02 | 21.4 | 0.83 | 0.22 | 0.21 | 8.23 | 40.64 |
| **Beijing** | 6.2 | 915.3 | 220.8 | 460.44 | 23.17 | 10.87 | 15.00 | 21.92 | 330.3 | 94.52 | 140.1 | 14.95 | 2670.28 | 16.35 | 81.73 | 49.80 |
| **Bondville** | 2.4 | 27.3 | 29.4 | 93.01 | 0.73 | 0.11 | 2.23 | 1.08 | 25.1 | 2.68 | 11.9 | 0.54 | 0.30 | 0.14 | 1.90 | 0.94 |
| **Dhaka** | 23.4 | 439.1 | 57.3 | 61.89 | 3.83 | 23.44 | 27.07 | 70.60 | 158.5 | 26.84 | 695.6 | 26.21 | 30.22 | 24.74 | 47.21 | 663.49 |
| **Halifax** | 1.6 | 13.4 | 34.6 | 1.95 | 0.20 | 0.48 | 0.93 | 0.32 | 5.2 | 1.16 | 9.3 | 0.16 | 0.08 | 0.01 | 1.20 | 1.01 |
| **Hanoi** | 32.2 | 567.4 | 50.6 | 27.26 | 2.34 | 0.97 | 1.34 | 82.96 | 150.1 | 9.04 | 1480.8 | 7.54 | 1.69 | 4.81 | 3.89 | 169.83 |
| **Ilorin** | 9.3 | 268.9 | 6.0 | 13.87 | 0.42 | 0.22 | 51.53 | 2.82 | 156.9 | 0.90 | 15.1 | 0.12 | 0.05 | 0.05 | 0.87 | 3.20 |
| **Kanpur** | 90.3 | 1934.1 | 99.0 | 462.42 | 3.33 | 1.59 | 33.50 | 6.89 | 154.7 | 6.89 | 124.9 | 19.02 | 12.08 | 40.44 | 3.34 | 293.24 |
| **Kelowna** | 3.2 | 21.7 | 3.4 | 4.00 | 0.32 | 0.29 | 0.61 | 0.45 | 15.7 | 0.63 | 2.5 | 0.16 | 0.05 | 0.02 | 0.53 | 0.29 |
| **Lethbridge** | 7.2 | 77.2 | 8.0 | 1.90 | 0.24 | 0.05 | 0.30 | 0.74 | 18.3 | 0.72 | 1.8 | 0.09 | 0.08 | 0.04 | 0.53 | 0.23 |
| **Manila** | 2.7 | 89.3 | 12.0 | 38.95 | 0.56 | 1.72 | 9.42 | 2.07 | 187.0 | 2.44 | 22.4 | 0.13 | 1.05 | 0.16 | 2.23 | 3.31 |
| **Pretoria** | 12.9 | 103.7 | 7.0 | 49.26 | 1.20 | 0.41 | 0.68 | 4.81 | 79.9 | 1.51 | 21.0 | 0.86 | 0.48 | 0.11 | 2.09 | 4.19 |
| **Rehovot** | 7.8 | 102.1 | 261.7 | 21.79 | 6.91 | 1.46 | 5.74 | 5.90 | 304.6 | 4.32 | 26.5 | 0.18 | 0.22 | 0.06 | 7.06 | 7.81 |
| **Sherbrooke** | 2.2 | 28.8 | 5.4 | 9.97 | 0.33 | 0.05 | 0.25 | 1.26 | 8.4 | 0.78 | 5.0 | 0.22 | 0.08 | 0.04 | 0.63 | 0.70 |
| **Singapore** | 7.2 | 134.7 | 36.5 | 22.93 | 0.94 | 15.05 | 0.63 | 4.91 | 41.9 | 6.40 | 147.8 | 0.30 | 0.70 | 0.10 | 3.01 | 1.87 |
| **Toronto** | 2.5 | 120.7 | 28.6 | 25.04 | 0.53 | 0.09 | 2.22 | 1.38 | 32.8 | 4.05 | 15.3 | 0.24 | 0.22 | 0.07 | 9.97 | 1.01 |

Table S5: Relative abundances of trace metals at SPARTAN sites as compared to the low-trace metal reference site of Mammoth Cave. Values shown in the table are unitless.

|  | **K** | **Mg** | **P** | **Ti** | **V** | **Cr** | **Mn** | **Fe** | **Cu** | **Zn** | **As** | **Se** | **Cd** | **Ba** | **Pb** |
| --- | --- | --- | --- | --- | --- | --- | --- | --- | --- | --- | --- | --- | --- | --- | --- |
| **Atlanta** | 0.4 | 0.4 | 1.9 | 1.2 | 0.2 | 3.5 | 0.4 | 0.6 | 1.0 | 1.1 | 2.1 | 2.0 | 0.3 | 1.7 | 1.2 |
| **Bandung** | 5.8 | 0.7 | 0.4 | 1.7 | 0.4 | 1.8 | 1.5 | 0.9 | 0.9 | 2.8 | 2.3 | 0.8 | 6.8 | 0.8 | 38.5 |
| **Beijing** | 12.9 | 6.3 | 2.7 | 11.1 | 3.0 | 3.7 | 13.0 | 4.7 | 7.3 | 12.0 | 27.1 | 243.8 | 69.7 | 7.0 | 45.9 |
| **Bondville** | 0.9 | 1.2 | 2.9 | 1.4 | 0.2 | 3.1 | 0.9 | 0.4 | 0.8 | 2.0 | 2.5 | 2.0 | 2.5 | 0.7 | 1.7 |
| **Buenos Aires** | 2.0 | 1.1 | 0.5 | 1.7 | 3.2 | 0.9 | 1.4 | 1.1 | 1.4 | 2.5 | 1.6 | 1.3 | 6.4 | 1.6 | 11.6 |
| **Dhaka** | 11.7 | 1.7 | 0.4 | 3.9 | 9.0 | 6.7 | 13.8 | 2.0 | 3.2 | 58.8 | 24.1 | 19.5 | 155.7 | 4.0 | 311.0 |
| **Halifax** | 0.5 | 0.6 | 0.0 | 0.2 | 0.3 | 0.3 | 0.2 | 0.1 | 0.2 | 0.4 | 0.5 | 0.3 | 0.0 | 0.2 | 0.5 |
| **Hanoi** | 17.3 | 3.0 | 0.7 | 5.2 | 2.7 | 1.9 | 43.6 | 3.4 | 3.8 | 139.1 | 30.9 | 10.9 | 90.1 | 2.3 | 156.8 |
| **Ilorin** | 4.8 | 0.6 | 0.1 | 0.9 | 0.8 | 39.9 | 2.5 | 2.2 | 0.3 | 1.5 | 0.8 | 0.5 | 1.2 | 0.3 | 4.7 |
| **Kanpur** | 40.8 | 2.7 | 6.1 | 5.5 | 2.8 | 16.2 | 5.4 | 2.0 | 2.4 | 14.1 | 58.2 | 38.3 | 273.0 | 1.3 | 232.7 |
| **Kelowna** | 0.5 | 0.1 | 0.0 | 0.3 | 0.2 | 0.3 | 0.2 | 0.2 | 0.2 | 0.2 | 0.7 | 0.1 | 0.1 | 0.2 | 0.3 |
| **Lethbridge** | 0.8 | 0.2 | 0.0 | 0.3 | 0.0 | 0.2 | 0.4 | 0.2 | 0.2 | 0.2 | 0.7 | 0.5 | 0.6 | 0.3 | 0.4 |
| **Manila** | 3.4 | 0.7 | 0.6 | 1.3 | 3.0 | 2.5 | 1.7 | 1.3 | 0.8 | 3.5 | 1.2 | 3.5 | 5.3 | 0.7 | 6.6 |
| **Pretoria** | 2.9 | 0.5 | 0.7 | 1.6 | 0.6 | 0.7 | 3.1 | 1.3 | 0.6 | 3.2 | 3.8 | 2.0 | 2.1 | 0.8 | 5.4 |
| **Rehovot** | 0.6 | 0.2 | 0.1 | 0.4 | 0.0 | 0.2 | 0.5 | 0.2 | 0.2 | 0.5 | 1.0 | 0.4 | 0.5 | 0.2 | 1.2 |
| **Sherbrooke** | 1.8 | 2.9 | 0.2 | 2.2 | 3.8 | 1.3 | 1.5 | 1.5 | 0.9 | 1.5 | 1.0 | 1.2 | 1.9 | 1.2 | 5.2 |
| **Singapore** | 4.6 | 0.9 | 0.2 | 1.6 | 48.7 | 0.4 | 4.2 | 1.1 | 1.5 | 13.0 | 1.8 | 2.6 | 2.5 | 1.2 | 3.9 |
| **Toronto** | 1.0 | 0.4 | 0.2 | 0.8 | 0.1 | 0.6 | 0.9 | 0.6 | 0.7 | 1.3 | 1.2 | 1.0 | 0.9 | 1.2 | 1.5 |

Table S6: PM_2.5_-relative elemental concentrations at SPARTAN sites compared to relative elemental concentrations at the low-trace metal reference site of Mammoth Cave. Values shown in the table are unitless.

|  | **K** | **Mg** | **P** | **Ti** | **V** | **Cr** | **Mn** | **Fe** | **Cu** | **Zn** | **As** | **Se** | **Cd** | **Ba** | **Pb** |
| --- | --- | --- | --- | --- | --- | --- | --- | --- | --- | --- | --- | --- | --- | --- | --- |
| **Atlanta** | 0.6 | 0.7 | 3.1 | 2.0 | 0.4 | 5.8 | 0.6 | 1.0 | 1.6 | 1.8 | 3.6 | 3.2 | 0.7 | 2.9 | 2.0 |
| **Bandung** | 3.3 | 0.4 | 0.2 | 1.0 | 0.2 | 1.0 | 0.9 | 0.5 | 0.5 | 1.6 | 1.3 | 0.5 | 3.6 | 0.5 | 21.8 |
| **Beijing** | 3.1 | 1.5 | 0.7 | 2.7 | 0.7 | 0.9 | 3.2 | 1.2 | 1.8 | 2.9 | 6.7 | 58.8 | 16.1 | 1.7 | 11.2 |
| **Bondville** | 2.1 | 3.0 | 7.3 | 3.4 | 0.4 | 7.8 | 2.2 | 1.1 | 2.1 | 4.9 | 6.2 | 4.9 | 6.0 | 1.7 | 4.3 |
| **Buenos Aires** | 3.0 | 1.6 | 0.8 | 2.5 | 4.7 | 1.4 | 2.0 | 1.7 | 2.1 | 3.6 | 2.4 | 1.9 | 8.9 | 2.4 | 17.2 |
| **Dhaka** | 3.4 | 0.5 | 0.1 | 1.1 | 2.6 | 1.9 | 4.0 | 0.6 | 0.9 | 17.0 | 7.1 | 5.6 | 42.6 | 1.2 | 90.1 |
| **Halifax** | 1.8 | 2.1 | 0.1 | 0.8 | 1.0 | 1.1 | 0.7 | 0.4 | 0.7 | 1.5 | 1.8 | 0.8 | 0.0 | 0.8 | 1.8 |
| **Hanoi** | 5.2 | 0.9 | 0.2 | 1.6 | 0.8 | 0.6 | 13.1 | 1.0 | 1.2 | 41.8 | 9.4 | 3.2 | 25.6 | 0.7 | 47.2 |
| **Ilorin** | 4.1 | 0.5 | 0.1 | 0.7 | 0.7 | 34.2 | 2.1 | 1.9 | 0.2 | 1.3 | 0.7 | 0.4 | 1.0 | 0.2 | 4.1 |
| **Kanpur** | 5.6 | 0.4 | 0.8 | 0.8 | 0.4 | 2.2 | 0.7 | 0.3 | 0.3 | 1.9 | 8.1 | 5.2 | 35.6 | 0.2 | 32.1 |
| **Kelowna** | 1.8 | 0.3 | 0.1 | 1.3 | 0.7 | 1.1 | 0.9 | 0.8 | 0.6 | 0.7 | 3.0 | 0.6 | 0.8 | 1.0 | 1.3 |
| **Lethbridge** | 1.7 | 0.5 | 0.1 | 0.7 | 0.1 | 0.5 | 0.8 | 0.5 | 0.5 | 0.5 | 1.5 | 1.1 | 1.4 | 0.6 | 1.0 |
| **Manila** | 3.1 | 0.7 | 0.6 | 1.2 | 2.7 | 2.3 | 1.6 | 1.2 | 0.7 | 3.2 | 1.2 | 3.2 | 4.6 | 0.7 | 6.0 |
| **Pretoria** | 2.3 | 0.4 | 0.5 | 1.3 | 0.4 | 0.5 | 2.4 | 1.0 | 0.5 | 2.5 | 3.0 | 1.5 | 1.6 | 0.6 | 4.2 |
| **Sherbrooke** | 1.6 | 0.4 | 0.2 | 1.1 | 0.1 | 0.4 | 1.2 | 0.5 | 0.5 | 1.2 | 2.5 | 0.9 | 1.0 | 0.5 | 3.0 |
| **Rehovot** | 1.7 | 2.6 | 0.2 | 2.0 | 3.5 | 1.2 | 1.4 | 1.4 | 0.8 | 1.4 | 0.9 | 1.1 | 1.7 | 1.1 | 4.8 |
| **Singapore** | 3.7 | 0.7 | 0.2 | 1.3 | 39.4 | 0.3 | 3.4 | 0.9 | 1.2 | 10.5 | 1.5 | 2.1 | 1.9 | 1.0 | 3.2 |
| **Toronto** | 2.0 | 0.9 | 0.4 | 0.0 | 0.0 | 1.3 | 1.8 | 1.2 | 1.6 | 2.7 | 2.5 | 2.2 | 1.7 | 2.6 | 3.2 |

Table S7: Full elemental breakdown of mean mass concentrations of trace metals in PM_10_ at SPARTAN sites. Mass concentrations of each trace metal are reported in ng/m^3^. Total PM_10_ mass concentrations are reported in µg/m^3^.

|  | **PM_10_** | **K** | **Mg** | **P** | **Ti** | **V** | **Cr** | **Mn** | **Fe** | **Cu** | **Zn** | **As** | **Se** | **Cd** | **Ba** | **Pb** |
| --- | --- | --- | --- | --- | --- | --- | --- | --- | --- | --- | --- | --- | --- | --- | --- | --- |
| **Bandung** | 43.7 | 583.9 | 106.1 | 38.2 | 9.4 | 1.5 | 5.3 | 9.1 | 280.3 | 8.5 | 40.2 | 1.6 | 1.2 | 1.1 | 7.8 | 43.4 |
| **Beijing** | 129.9 | 1448.6 | 699.6 | 195.5 | 40.1 | 7.3 | 14.6 | 53.7 | 1138.0 | 57.1 | 161.6 | 13.3 | 91.8 | 15.5 | 57.5 | 61.9 |
| **Buenos Aires** | 23.1 | 250.3 | 131.0 | 50.0 | 6.2 | 3.4 | 2.9 | 6.5 | 270.9 | 9.3 | 29.6 | 0.6 | 0.5 | 0.3 | 9.5 | 12.4 |
| **Dhaka** | 118.3 | 1365.6 | 339.5 | 59.6 | 44.1 | 17.7 | 21.0 | 67.6 | 1040.9 | 25.5 | 1062.6 | 16.7 | 15.6 | 18.5 | 31.6 | 486.5 |
| **Halifax** | 8.2 | 55.0 | 37.2 | 2.5 | 1.5 | 0.2 | 0.6 | 0.9 | 48.3 | 4.7 | 5.0 | 0.2 | 0.2 | 0.0 | 5.1 | 1.0 |
| **Hanoi** | 107.1 | 1558.8 | 226.8 | 67.9 | 9.4 | 3.1 | 3.4 | 92.3 | 470.5 | 15.6 | 1262.9 | 8.4 | 3.0 | 4.4 | 9.3 | 151.5 |
| **Ilorin** | 29.5 | 453.1 | 61.6 | 21.2 | 2.8 | 0.8 | 60.1 | 9.1 | 287.5 | 1.8 | 24.5 | 0.3 | 0.2 | 0.1 | 2.2 | 6.7 |
| **Kanpur** | 174.0 | 3855.9 | 418.9 | 682.8 | 22.9 | 4.4 | 341.0 | 27.1 | 681.0 | 18.9 | 215.3 | 21.0 | 14.1 | 14.0 | 14.8 | 295.5 |
| **Kelowna** | 11.2 | 75.0 | 22.3 | 3.0 | 3.6 | 0.2 | 1.2 | 2.2 | 86.3 | 1.3 | 0.4 | 0.2 | 0.0 | 0.0 | 4.2 | 0.3 |
| **Lethbridge** | 9.9 | 62.00 | 74.77 | 10.17 | 2.07 | 0.20 | 0.20 | 6.57 | 145.55 | 2.87 | 6.20 | 0.13 | 0.17 | 0.00 | 6.23 | 0.33 |
| **Manila** | 33.7 | 435.8 | 177.4 | 57.2 | 10.6 | 3.9 | 3.8 | 10.8 | 256.9 | 5.7 | 58.3 | 0.6 | 1.5 | 0.4 | 8.7 | 12.8 |
| **Pretoria** | 31.2 | 344.0 | 100.4 | 66.6 | 6.8 | 1.9 | 3.4 | 17.9 | 368.6 | 7.0 | 79.4 | 1.3 | 0.8 | 0.1 | 7.8 | 6.7 |
| **Rehovot** | 36.9 | 254.0 | 413.6 | 31.9 | 7.3 | 3.7 | 2.4 | 7.7 | 380.4 | 9.0 | 22.5 | 0.4 | 0.5 | 0.1 | 9.1 | 6.2 |
| **Sherbrooke** | 12.0 | 75.0 | 31.9 | 0.8 | 2.1 | 0.1 | 0.9 | 2.8 | 68.7 | 1.5 | 5.5 | 0.2 | 0.2 | 0.0 | 1.8 | 1.0 |
| **Toronto** | 15.7 | 75.0 | 81.3 | 2.5 | 3.8 | 0.2 | 0.4 | 5.0 | 185.6 | 11.9 | 12.5 | 0.4 | 0.3 | 0.0 | 12.9 | 1.4 |

Table S8: Elemental breakdown by percentage of PM_2.5_ accounted for by each trace element. PM_2.5_ concentrations are reported in µg/m^3^. All other values are element-specific percentages of each site’s PM_2.5_ concentration.

|  | **PM2.5** | **K** | **Mg** | **P** | **Ti** | **V** | **Cr** | **Mn** | **Fe** | **Cu** | **Zn** | **As** | **Se** | **Cd** | **Ba** | **Pb** |
| --- | --- | --- | --- | --- | --- | --- | --- | --- | --- | --- | --- | --- | --- | --- | --- | --- |
| **Mammoth Cave** | 14.2 | 0.5268 | 0.1972 | 0.3923 | 0.0071 | 0.0055 | 0.0085 | 0.0130 | 0.5859 | 0.0259 | 0.0599 | 0.0018 | 0.0020 | 0.0004 | 0.0220 | 0.0063 |
| **Atlanta** | 8.6 | 0.3233 | 0.1337 | 1.2244 | 0.0144 | 0.0021 | 0.0488 | 0.0083 | 0.5988 | 0.0427 | 0.1081 | 0.0065 | 0.0063 | 0.0002 | 0.0629 | 0.0126 |
| **Bandung** | 25.1 | 1.7215 | 0.0729 | 0.0952 | 0.0069 | 0.0012 | 0.0084 | 0.0112 | 0.3116 | 0.0131 | 0.0944 | 0.0024 | 0.0009 | 0.0013 | 0.0102 | 0.1379 |
| **Beijing** | 58.1 | 1.6568 | 0.3052 | 0.2601 | 0.0192 | 0.0040 | 0.0076 | 0.0410 | 0.6793 | 0.0460 | 0.1747 | 0.0123 | 0.1160 | 0.0057 | 0.0375 | 0.0711 |
| **Bondville** | 5.7 | 1.1246 | 0.5982 | 2.8474 | 0.0244 | 0.0023 | 0.0661 | 0.0279 | 0.6491 | 0.0546 | 0.2930 | 0.0114 | 0.0096 | 0.0021 | 0.0377 | 0.0270 |
| **Buenos Aires** | 9.6 | 1.5844 | 0.3167 | 0.3156 | 0.0177 | 0.0258 | 0.0115 | 0.0263 | 0.9792 | 0.0539 | 0.2167 | 0.0045 | 0.0038 | 0.0031 | 0.0529 | 0.1091 |
| **Dhaka** | 49 | 1.7878 | 0.0978 | 0.0469 | 0.0080 | 0.0142 | 0.0163 | 0.0518 | 0.3422 | 0.0239 | 1.0171 | 0.0129 | 0.0110 | 0.0150 | 0.0256 | 0.5709 |
| **Halifax** | 4.2 | 0.9524 | 0.4238 | 0.0262 | 0.0060 | 0.0055 | 0.0093 | 0.0086 | 0.2571 | 0.0181 | 0.0881 | 0.0033 | 0.0017 | 0.0000 | 0.0179 | 0.0117 |
| **Hanoi** | 47.1 | 2.7469 | 0.1794 | 0.0777 | 0.0111 | 0.0045 | 0.0048 | 0.1700 | 0.5992 | 0.0299 | 2.5028 | 0.0172 | 0.0064 | 0.0090 | 0.0151 | 0.2994 |
| **Ilorin** | 16.6 | 2.1422 | 0.0934 | 0.0307 | 0.0053 | 0.0037 | 0.2889 | 0.0272 | 1.0988 | 0.0057 | 0.0777 | 0.0013 | 0.0008 | 0.0004 | 0.0054 | 0.0257 |
| **Kanpur** | 102.8 | 2.9641 | 0.0724 | 0.3308 | 0.0054 | 0.0021 | 0.0189 | 0.0096 | 0.1634 | 0.0085 | 0.1162 | 0.0149 | 0.0103 | 0.0125 | 0.0039 | 0.2036 |
| **Kelowna** | 3.5 | 0.9743 | 0.0686 | 0.0457 | 0.0091 | 0.0040 | 0.0091 | 0.0117 | 0.4857 | 0.0157 | 0.0400 | 0.0054 | 0.0011 | 0.0003 | 0.0214 | 0.0083 |
| **Lethbridge** | 6.2 | 0.9081 | 0.1032 | 0.0242 | 0.0048 | 0.0005 | 0.0040 | 0.0110 | 0.2984 | 0.0123 | 0.0306 | 0.0027 | 0.0023 | 0.0005 | 0.0137 | 0.0061 |
| **Manila** | 15.4 | 1.6448 | 0.1318 | 0.2234 | 0.0084 | 0.0151 | 0.0193 | 0.0206 | 0.7221 | 0.0190 | 0.1909 | 0.0021 | 0.0062 | 0.0016 | 0.0150 | 0.0382 |
| **Pretoria** | 18.3 | 1.2022 | 0.0809 | 0.2049 | 0.0091 | 0.0024 | 0.0044 | 0.0316 | 0.5749 | 0.0125 | 0.1503 | 0.0055 | 0.0030 | 0.0005 | 0.0139 | 0.0267 |
| **Sherbrooke** | 5.7 | 0.8474 | 0.0877 | 0.0754 | 0.0079 | 0.0005 | 0.0035 | 0.0161 | 0.2930 | 0.0128 | 0.0737 | 0.0046 | 0.0018 | 0.0004 | 0.0102 | 0.0189 |
| **Rehovot** | 15.4 | 0.8779 | 0.5188 | 0.0740 | 0.0142 | 0.0192 | 0.0101 | 0.0183 | 0.8019 | 0.0209 | 0.0831 | 0.0017 | 0.0022 | 0.0006 | 0.0240 | 0.0301 |
| **Singapore** | 17.5 | 1.9709 | 0.1383 | 0.0766 | 0.0095 | 0.2167 | 0.0027 | 0.0443 | 0.5137 | 0.0318 | 0.6291 | 0.0027 | 0.0041 | 0.0007 | 0.0217 | 0.0202 |
| **Toronto** | 6.7 | 1.0597 | 0.1791 | 0.1448 | 0.0124 | 0.0013 | 0.0107 | 0.0237 | 0.6940 | 0.0404 | 0.1597 | 0.0046 | 0.0043 | 0.0006 | 0.0570 | 0.0206 |

# Supplemental References

(1) Chow, J. C.; Watson, J. G.; Lu, Z.; Lowenthal, D. H.; Frazier, C. A.; Solomon, P. A.; Thuillier, R. H.; Magliano, K. Descriptive Analysis of PM2.5 and PM10 at Regionally Representative Locations during SJVAQS/AUSPEX. *Atmos. Environ.* **1996**, *30* (12), 2079–2112. https://doi.org/http://dx.doi.org/10.1016/1352-2310(95)00402-5.

(2) Yao, X.; Chan, C. K.; Fang, M.; Cadle, S.; Chan, T.; Mulawa, P.; He, K.; Ye, B. The Water-Soluble Ionic Composition of PM2.5 in Shanghai and Beijing, China. *Atmos. Environ.* **2002**, *36* (26), 4223–4234. https://doi.org/http://dx.doi.org/10.1016/S1352-2310(02)00342-4.

(3) Munchak, L. A.; Schichtel, B. A.; Sullivan, A. P.; Holden, A. S.; Kreidenweis, S. M.; Malm, W. C.; Collett, J. L. Development of Wildland Fire Particulate Smoke Marker to Organic Carbon Emission Ratios for the Conterminous United States. *Atmos. Environ.* **2011**, *45* (2), 395–403. https://doi.org/10.1016/j.atmosenv.2010.10.006.

(4) Hsu, S.-C.; Liu, S. C.; Huang, Y.-T.; Chou, C. C. K.; Lung, S. C. C.; Liu, T.-H.; Tu, J.-Y.; Tsai, F. Long-Range Southeastward Transport of Asian Biosmoke Pollution: Signature Detected by Aerosol Potassium in Northern Taiwan. *J. Geophys. Res. Atmos.* **2009**, *114* (D14), D14301. https://doi.org/10.1029/2009JD011725.

(5) Snider, G.; Weagle, C. L.; Murdymootoo, K. K.; Ring, A.; Ritchie, Y.; Stone, E.; Walsh, A.; Akoshile, C.; Anh, N. X.; Balasubramanian, R.; et al. Variation in Global Chemical Composition of PM_2.5_: Emerging Results from SPARTAN. *Atmos. Chem. Phys.* **2016**, *16* (15), 9629–9653. https://doi.org/10.5194/acp-16-9629-2016.

(6) Zhang, W.-J.; Sun, Y.-L.; Zhuang, G.-S.; Xu, D.-Q. Characteristics and Seasonal Variations of PM2.5, PM10, and TSP Aerosol in Beijing. *Biomed. Environ. Sci.* **2006**, *19* (6), 461–468.

(7) Malm, W. C.; Sisler, J. F.; Huffman, D.; Eldred, R. A.; Cahill, T. A. Spatial and Seasonal Trends in Particle Concentration and Optical Extinction in the United States. *J. Geophys. Res.* **1994**, *99* (D1), 1347–1370.

(8) Wang, Y.; Zhuang, G.; Zhang, X.; Huang, K.; Xu, C.; Tang, A.; Chen, J.; An, Z. The Ion Chemistry, Seasonal Cycle, and Sources of PM2.5 and TSP Aerosol in Shanghai. *Atmos. Environ.* **2006**, *40* (16), 2935–2952. https://doi.org/10.1016/j.atmosenv.2005.12.051.

(9) Taylor, S. R.; McLennan, S. M. The Geochemical Evolution of the Continental Crust. *Rev. Geophys.* **1995**, *33* (2), 241–265. https://doi.org/10.1029/95RG00262.

(10) Pacyna, J. M.; Pacyna, E. G. An Assessment of Global and Regional Emissions of Trace Metals to the Atmosphere from Anthropogenic Sources Worldwide. *Environ. Rev.* **2001**, *9* (4), 269–298. https://doi.org/10.1139/a01-012.

(11) Laden, F.; Neas, L. M.; Dockery, D. W.; Schwartz, J. Association of Fine Particulate Matter from Different Sources with Daily Mortality in Six U.S. Cities. *Environ. Health Perspect.* **2000**, *108* (10), 941–947. https://doi.org/10.2307/3435052.

(12) Gibson, M. D.; Haelssig, J.; Pierce, J. R.; Parrington, M.; Franklin, J. E.; Hopper, J. T.; Li, Z.; Ward, T. J. A Comparison of Four Receptor Models Used to Quantify the Boreal Wildfire Smoke Contribution to Surface PM_2.5_ in Halifax, Nova Scotia during the BORTAS-B Experiment. *Atmos. Chem. Phys.* **2015**, *15* (2), 815–827. https://doi.org/10.5194/acp-15-815-2015.

(13) Gibson, M. D.; Pierce, J. R.; Waugh, D.; Kuchta, J. S.; Chisholm, L.; Duck, T. J.; Hopper, J. T.; Beauchamp, S.; King, G. H.; Franklin, J. E.; et al. Identifying the Sources Driving Observed PM2.5 Temporal Variability over Halifax, Nova Scotia, during BORTAS-B. *Atmos. Chem. Phys.* **2013**, *13* (14), 7199–7213. https://doi.org/10.5194/acp-13-7199-2013.

(14) Ueki, T. Vanadium in the Environment and Its Bioremediation. In *Plants, Pollutants and Remediation*; Öztürk, M., Ashraf, M., Aksoy, A., Ahmad, A. M. S., Hakeem, R. K., Eds.; Springer Netherlands: Dordrecht, 2015; pp 13–26. https://doi.org/10.1007/978-94-017-7194-8_2.

(15) Chillrud, S. N.; Grass, D.; Ross, J. M.; Coulibaly, D.; Slavkovich, V.; Epstein, D.; Sax, S. N.; Pederson, D.; Johnson, D.; Spengler, J. D.; et al. Steel Dust in the New York City Subway System as a Source of Manganese, Chromium, and Iron Exposures for Transit Workers. *J. Urban Health* **2005**, *82* (1), 33–42. https://doi.org/10.1093/jurban/jti006.

(16) Lippmann, M. Toxicological and Epidemiological Studies of Cardiovascular Effects of Ambient Air Fine Particulate Matter (PM2.5) and Its Chemical Components: Coherence and Public Health Implications. *Crit. Rev. Toxicol.* **2014**, *44* (4), 299–347. https://doi.org/10.3109/10408444.2013.861796.

(17) Habibu, S.; Koki, I. B.; Tukur, A. I.; Gumel, S. M.; Ado, A.; Ladan, M.; Muhammad, A. A. A Reviewon Industrial Effluents as Major Sources of Water Pollution in Nigeria Riverbank Filtration and Surface Water Pollution View Project Electrochemical Sensor View Project A Review on Industrial Effluents as Major Sources of Water Pollution in Nigeria. *Chem. J.* **2015**, *1* (5), 159–164.

(18) Pekney, N. J.; Davidson, C. I.; Robinson, A.; Zhou, L.; Hopke, P.; Eatough, D.; Rogge, W. F. Major Source Categories for PM2.5 in Pittsburgh Using PMF and UNMIX. *Aerosol Sci. Technol.* **2006**, *40* (10), 910–924. https://doi.org/10.1080/02786820500380271.

(19) Das, R.; Khezri, B.; Srivastava, B.; Datta, S.; Sikdar, P. K.; Webster, R. D.; Wang, X. Trace Element Composition of PM2.5 and PM10 from Kolkata – a Heavily Polluted Indian Metropolis. *Atmos. Pollut. Res.* **2015**, *6* (5), 742–750. https://doi.org/http://dx.doi.org/10.5094/APR.2015.083.

(20) Dai, Q.-L.; Bi, X.-H.; Wu, J.-H.; Zhang, Y.-F.; Wang, J.; Xu, H.; Yao, L.; Jiao, L.; Feng, Y.-C. Characterization and Source Identification of Heavy Metals in Ambient PM10 and PM2.5 in an Integrated Iron and Steel Industry Zone Compared with a Background Site. *Aerosol Air Qual. Res.* **2015**, *15* (3), 875–887. https://doi.org/10.4209/aaqr.2014.09.0226.

(21) Kong, S.; Han, B.; Bai, Z.; Chen, L.; Shi, J.; Xu, Z. Receptor Modeling of PM2.5, PM10 and TSP in Different Seasons and Long-Range Transport Analysis at a Coastal Site of Tianjin, China. *Sci. Total Environ.* **2010**, *408* (20), 4681–4694. https://doi.org/10.1016/j.scitotenv.2010.06.005.

(22) Zhao, J.; Lewinski, N.; Riediker, M. Physico-Chemical Characterization and Oxidative Reactivity Evaluation of Aged Brake Wear Particles. *Aerosol Sci. Technol.* **2015**, *49* (2), 65–74. https://doi.org/10.1080/02786826.2014.998363.

(23) Begum, B. A.; Biswas, S. K.; Markwitz, A.; Hopke, P. K. Identification of Sources of Fine and Coarse Particulate Matter in Dhaka, Bangladesh. *Aerosol Air Qual. Res.* **2010**, *10*, 345–353. https://doi.org/10.4209/aaqr.2009.12.0082.

(24) Councell, T. B.; Duckenfield, K. U.; Landa, E. R.; Callender, E. Tire-Wear Particles as a Source of Zinc to the Environment. *Environ. Sci. Technol.* **2004**, *38* (15), 4206–4214. https://doi.org/10.1021/es034631f.

(25) Harrison, R. M.; Jones, A. M.; Gietl, J.; Yin, J.; Green, D. C. Estimation of the Contributions of Brake Dust, Tire Wear, and Resuspension to Nonexhaust Traffic Particles Derived from Atmospheric Measurements. *Environ. Sci. Technol.* **2012**, *46* (12), 6523–6529. https://doi.org/10.1021/es300894r.

(26) Zheng, N.; Liu, J.; Wang, Q.; Liang, Z. Health Risk Assessment of Heavy Metal Exposure to Street Dust in the Zinc Smelting District, Northeast of China. *Sci. Total Environ.* **2010**, *408* (4), 726–733. https://doi.org/10.1016/j.scitotenv.2009.10.075.

(27) Dan, M.; Zhuang, G.; Li, X.; Tao, H.; Zhuang, Y. The Characteristics of Carbonaceous Species and Their Sources in PM2.5 in Beijing. *Atmos. Environ.* **2004**, *38* (21), 3443–3452. https://doi.org/http://dx.doi.org/10.1016/j.atmosenv.2004.02.052.

(28) Garcia-Aleix, J. R.; Delgado-Saborit, J. M.; Verdú-Martin, G.; Amigó-Descarrega, J. M.; Esteve-Cano, V. Trends in Arsenic Levels in PM10 and PM2.5 Aerosol Fractions in an Industrialized Area. *Environ. Sci. Pollut. Res.* **2013**, *21* (1), 695–703. https://doi.org/10.1007/s11356-013-1950-0.

(29) Pacyna, E. G.; Pacyna, J. M.; Fudala, J.; Strzelecka-Jastrzab, E.; Hlawiczka, S.; Panasiuk, D.; Nitter, S.; Pregger, T.; Pfeiffer, H.; Friedrich, R. Current and Future Emissions of Selected Heavy Metals to the Atmosphere from Anthropogenic Sources in Europe. *Atmos. Environ.* **2007**, *41* (38), 8557–8566. https://doi.org/10.1016/j.atmosenv.2007.07.040.

(30) Okuda, T.; Kato, J.; Mori, J.; Tenmoku, M.; Suda, Y.; Tanaka, S.; He, K.; Ma, Y.; Yang, F.; Yu, X.; et al. Daily Concentrations of Trace Metals in Aerosols in Beijing, China, Determined by Using Inductively Coupled Plasma Mass Spectrometry Equipped with Laser Ablation Analysis, and Source Identification of Aerosols. *Sci. Total Environ.* **2004**, *330* (1–3), 145–158. https://doi.org/10.1016/j.scitotenv.2004.04.010.

(31) Hughes, M. F. *Arsenic Toxicity and Potential Mechanisms of Action*; 2002; Vol. 133. https://doi.org/10.1016/S0378-4274(02)00084-X.

(32) IARC. *Air Pollution and Cancer, IARC Publication No. 161*; Lyon, France, 2013.

(33) Duan, J.; Tan, J. Atmospheric Heavy Metals and Arsenic in China: Situation, Sources and Control Policies. *Atmos. Environ.* **2013**, *74*, 93–101. https://doi.org/http://dx.doi.org/10.1016/j.atmosenv.2013.03.031.

(34) Eldred, R. A.; Cahill, T. A.; Flocchini, R. G. Composition of PM2.5 and PM10 Aerosols in the IMPROVE Network. *J. Air Waste Manage. Assoc.* **1997**, *47* (2), 194–203. https://doi.org/10.1080/10473289.1997.10464422.

(35) Godt, J.; Scheidig, F.; Grosse-Siestrup, C.; Esche, V.; Brandenburg, P.; Reich, A.; Groneberg, D. A. The Toxicity of Cadmium and Resulting Hazards for Human Health. *J. Occup. Med. Toxicol.* **2006**, *1* (1), 1–6. https://doi.org/10.1186/1745-6673-1-22.

(36) Martin, S.; Griswold, W. Human Health Effects of Heavy Metals. *Environ. Sci. Technol. briefs citizens* **2009**, *15*, 1–6.

(37) Monaci, F.; Bargagli, R. Barium and Other Trace Metals as Indicators of Vehicle Emissions. *Water. Air. Soil Pollut.* **1997**, *100* (1), 89–98. https://doi.org/10.1023/A:1018318427017.

(38) Fang, T.; Guo, H.; Verma, V.; Peltier, R. E.; Weber, R. J. PM_2.5_ Water-Soluble Elements in the Southeastern United States: Automated Analytical Method Development, Spatiotemporal Distributions, Source Apportionment, and Implications for Heath Studies. *Atmos. Chem. Phys.* **2015**, *15* (20), 11667–11682. https://doi.org/10.5194/acp-15-11667-2015.

(39) Secrest, M. H.; Schauer, J. J.; Carter, E. M.; Lai, A. M.; Wang, Y.; Shan, M.; Yang, X.; Zhang, Y.; Baumgartner, J. The Oxidative Potential of PM2.5 Exposures from Indoor and Outdoor Sources in Rural China. *Sci. Total Environ.* **2016**. https://doi.org/10.1016/j.scitotenv.2016.06.231.

(40) Klee, R. J.; Graedel, T. E. Elemental Cycles: A Status Report on Human or Natural Dominance. *Annu. Rev. Environ. Resour.* **2004**, *29* (1), 69–107. https://doi.org/10.1146/annurev.energy.29.042203.104034.

(41) Hu, X.; Zhang, Y.; Ding, Z.; Wang, T.; Lian, H.; Sun, Y.; Wu, J. Bioaccessibility and Health Risk of Arsenic and Heavy Metals (Cd, Co, Cr, Cu, Ni, Pb, Zn and Mn) in TSP and PM2.5 in Nanjing, China. *Atmos. Environ.* **2012**, *57*, 146–152. https://doi.org/10.1016/j.atmosenv.2012.04.056.

(42) Song, Y.; Xie, S.; Zhang, Y.; Zeng, L.; Salmon, L. G.; Zheng, M. Source Apportionment of PM2.5 in Beijing Using Principal Component Analysis/Absolute Principal Component Scores and UNMIX. *Sci. Total Environ.* **2006**, *372* (1), 278–286. https://doi.org/10.1016/j.scitotenv.2006.08.041.

(43) Lestari, P.; Mauliadi, Y. D. Source Apportionment of Particulate Matter at Urban Mixed Site in Indonesia Using PMF. *Atmos. Environ.* **2009**, *43* (10), 1760–1770. https://doi.org/http://dx.doi.org/10.1016/j.atmosenv.2008.12.044.

(44) Gidlow, D. A. Lead Toxicity. *Occup. Med. (Chic. Ill).* **2004**, *54* (2), 76–81. https://doi.org/10.1093/occmed/kqh019.

(45) USEPA. *National Ambient Air Quality Standards for Lead*; Washington, DC, 2008.

(46) EPA. National Ambient Air Quality Standards (NAAQS).

(47) IMPROVE. Reconstructing Light Extinction from Aerosol Measurements.

(48) USEPA. Chemical Speciation Network Database.

(49) Weagle, C. L.; Snider, G.; Li, C.; Van Donkelaar, A.; Philip, S.; Bissonnette, P.; Burke, J.; Jackson, J.; Latimer, R.; Stone, E.; et al. Global Sources of Fine Particulate Matter: Interpretation of PM2.5 Chemical Composition Observed by SPARTAN Using a Global Chemical Transport Model. *Environ. Sci. Technol.* **2018**, *52* (20), 11670–11681. https://doi.org/10.1021/acs.est.8b01658.

**Author Contributions Statement**

All listed authors of this work have contributed substantially to this research in some capacity, and have approved this manuscript. Contributions were as such:

- Core SPARTAN team (data collection, data analysis, experimental design, drafting of manuscript):
- JM, GS, CLW, BW, PB, ES, RVM, MB, YR
- SPARTAN site principal investigators and site operators (data collection)
  - IA, CA, NXA, RB, CC, JD, RMG, KH, NL, PL, YL, FJ, KSJ, AM, LKN, EJQ, AS, SNT, QZ
- SPARTAN network partners (experimental design)
  - JRB, AC, BNH, RK, JVM, BS, CW
- Laboratory research partners (data collection and analysis)
  - GG, JSK, MDG

1. *Corresponding author email: [jamcneill@dal.ca](mailto:jamcneill@dal.ca) or [rvmartin@wustl.edu](mailto:rvmartin@wustl.edu), phone: 314-935-2183

   *Affiliations*

   Department of Chemistry, Dalhousie University, Halifax, Canada [↑](#footnote-ref-1)
2. Department of Energy, Environmental and Chemical Engineering, Washington University in St. Louis, St. Louis, MO, USA [↑](#footnote-ref-2)
3. Department of Physics and Atmospheric Science, Dalhousie University, Halifax, Canada [↑](#footnote-ref-3)
4. Environment and Climate Change Canada, Downsview, Ontario, Canada & Kelowna, British Columbia, Canada [↑](#footnote-ref-4)
5. Department of Physics, University of Ilorin, Ilorin, Nigeria [↑](#footnote-ref-5)
6. Institute of Geophysics, Vietnam Academy of Science and Technology, Hanoi, Vietnam [↑](#footnote-ref-6)
7. Department of Civil and Environmental Engineering, National University of Singapore [↑](#footnote-ref-7)
8. Department of Public Health Sciences, University of Toronto, Toronto, Ontario, Canada M5S 1A8 [↑](#footnote-ref-8)
9. Department of Geography and Environment, University of Lethbridge, Lethbridge, Alberta, Canada [↑](#footnote-ref-9)
10. Health Effects Institute, 75 Federal Street Suite 1400, Boston, MA 02110-1817, USA [↑](#footnote-ref-10)
11. School of Environment, Tsinghua University, Beijing, China [↑](#footnote-ref-11)
12. Department of Civil and Resource Engineering, Dalhousie University, Halifax, Canada [↑](#footnote-ref-12)
13. Council for Scientific and Industrial Research (CSIR), Pretoria, South Africa [↑](#footnote-ref-13)
14. Unit for Environmental Sciences and Management, North-West University, Potchefstroom, South Africa [↑](#footnote-ref-14)
15. Department of Geography, Geo-informatics and Meteorology, University of Pretoria, Pretoria, South Africa [↑](#footnote-ref-15)
16. Department of Earth System Science, Tsinghua University, Beijing, China [↑](#footnote-ref-16)
17. Earth Science Division, NASA Goddard Space Flight Center, Greenbelt, Maryland, USA [↑](#footnote-ref-17)
18. Department of Community Health and Epidemiology, Dalhousie University, Halifax, Canada [↑](#footnote-ref-18)
19. Manila Observatory, Ateneo de Manila University campus, Quezon City, Philippines [↑](#footnote-ref-19)
20. Faculty of Civil and Environmental Engineering, Bandung Institute of Technology (ITB), JL. Ganesha No.10, Bandung 40132, Indonesia [↑](#footnote-ref-20)
21. Rollins School of Public Health, Emory University, 1518 Clifton Road NE, Atlanta, GA 30322, United States [↑](#footnote-ref-21)
22. Department of Chemistry, Faculty of Science, University of Dhaka, Dhaka - 1000, Bangladesh [↑](#footnote-ref-22)
23. Department of Physics and Joint Center for Earth Systems Technology, University of Maryland, Baltimore County, Baltimore, Maryland, USA [↑](#footnote-ref-23)
24. Center for Environmental Science and Engineering, Indian Institute of Technology Kanpur, India [↑](#footnote-ref-24)
25. Department of Architecture, Massachusetts Institute of Technology, Cambridge, MA, 02139, USA [↑](#footnote-ref-25)
26. UNIDEF (CITEDEF-CONICET) Juan B. de la Salle 4397 – B1603ALO Villa Martelli, Buenos Aires, Argentina [↑](#footnote-ref-26)
27. Cooperative Institute for Research in the Atmosphere, Colorado State University, Colorado, USA [↑](#footnote-ref-27)
28. Center for Global Change Science, Massachusetts Institute of Technology, Cambridge, MA, 02139, USA [↑](#footnote-ref-28)
29. School of Population and Public Health, University of British Columbia, Vancouver, British Columbia, Canada [↑](#footnote-ref-29)
30. Department of Civil and Resource Engineering, Dalhousie University, Halifax, Canada [↑](#footnote-ref-30)
31. AirPhoton, LLC., Baltimore, Maryland, USA [↑](#footnote-ref-31)
32. Department of Earth and Planetary Sciences, Weizmann Institute, Rehovot 76100, Israel [↑](#footnote-ref-32)
